# Identification of Receptor Binding Proteins of *Yersinia* Phage φR1-37 and Enterocoliticin That Use the Same Bacterial Surface Receptor

**DOI:** 10.3390/v18030291

**Published:** 2026-02-27

**Authors:** Mikael Skurnik, Rahime Tetik, Muhammad Suleman Qasim, Jana Sachsenröder, Ralf Dieckmann, Carlos G. Leon-Velarde, Göran Widmalm, Eckhard Strauch, Arnab Bhattacharjee

**Affiliations:** 1 Human Microbiome Research Program, Department of Bacteriology and Immunology, Faculty of Medicine, University of Helsinki, 00014 Helsinki, Finland; muhammad.qasim@helsinki.fi (M.S.Q.); arnab.bhattacharjee@helsinki.fi (A.B.); 2 Department Biological Safety, German Federal Institute for Risk Assessment, 10589 Berlin, Germany; ra_rahime@yahoo.de (R.T.); jana.sachsenroeder@bfr.bund.de (J.S.); dieckmannr@rki.de (R.D.); eckhard.strauch@bfr.bund.de (E.S.); 3 RNAcious Laboratory, Molecular and Integrative Biosciences Research Programme, Faculty of Biological and Environmental Sciences, University of Helsinki, 00014 Helsinki, Finland; 4 Department 1—Infectious Diseases, Robert Koch Institute, 13353 Berlin, Germany; 5 Laboratory Services Division, University of Guelph, Guelph, ON N1H 8J7, Canada; cleonvel@uoguelph.ca; 6 Arrhenius Laboratory, Department of Chemistry, Stockholm University, S-106 91 Stockholm, Sweden; goran.widmalm@su.se; 7 Drug Research Program, Division of Pharmaceutical Biosciences, Faculty of Pharmacy, University of Helsinki, 00014 Helsinki, Finland

**Keywords:** bacteriophage, tailocin, enterocoliticin, *Yersinia enterocolitica*, receptor binding protein, phage tail fiber, Alphafold3, CarbBuilder

## Abstract

The bacterium *Yersinia enterocolitica* serotype O:3 is targeted by two distinct agents, the bacteriophage φR1-37 and the bacteriocin-like enterocoliticin (a tailocin), which both utilize the lipopolysaccharide (LPS) outer core (OC) hexasaccharide as their primary host receptor. In order to understand this convergent recognition mechanism, we first characterized the enterocoliticin system, reporting the complete sequence of its large, biosynthetic gene cluster. Most of the 42 predicted gene products were functionally annotated by homology to known gene products. We then focused on identifying the receptor-binding proteins (RBPs) responsible for host attachment of both agents in order to elucidate a possible shared mechanism of binding. For phage φR1-37, the receptor binding complex was identified as the inseparable Gp298 tail fiber protein and its Gp297 trimerization chaperone, confirming its function as the RBP. Based on sequence identity with Gp298, the Orf39 gene product of the enterocoliticin cluster was predicted to be its corresponding RBP. An analytical comparison of the predicted RBPs revealed a highly conserved homologous region spanning 80–85 amino acid residues, which presents the only structural explanation for their identical receptor specificity. To resolve the binding mechanism, we generated high-confidence trimeric structural models for the Gp298 and Orf39 proteins using AlphaFold3-multimer. These models validated the high structural similarity of the RBP domains, despite global dissimilarity of the complete trimeric structures. Further docking simulations with a pentasaccharide ligand (generated by CarbBuilder) provided suggestive molecular models for the protein-carbohydrate interactions within the OC region. Intriguingly, a database search using the identified binding site motif revealed their wide and diverse presence in various phage tail proteins, suggesting that this motif is a specialized, common structure for carbohydrate recognition. This work identifies a conserved, novel sugar-binding motif as the molecular basis of host recognition for these key anti-*Yersinia* biologics.

## 1. Introduction

Genus *Yersinia* is a member of the order *Enterobacteriales,* family *Yersiniaceae* [1], and includes three species pathogenic to humans: *Yersinia pestis*, the causative agent of bubonic plague, and *Y. enterocolitica* and *Y. pseudotuberculosis* that usually cause gastrointestinal infections [2].

Due to increasing antibiotic resistance and negligent discovery of new antibiotics based on their potential as anti-infectious agents, phage therapy has regained attraction [3,4]. Phages are present almost everywhere, including water, soil and air. They can survive in all habitats where bacteria can survive. The discovery of bacteriophages (phages) specific for the genus *Yersinia* dates to 1925, when Felix d’Herelle isolated phages against *Yersinia pestis*, the causative agent of bubonic plague, and used the phages for the first time as an anti-plague agent on patients [5]. Since then, numerous yersiniophages have been isolated, classified and categorized [5,6,7]. A much studied *Yersinia* phage is φR1-37, a myovirus that was isolated based on its ability to infect the *Y. enterocolitica* serotype O:3 lipopolysaccharide (LPS) mutant strain YeO3-R1, an O-polysaccharide (OPS) lacking derivative of wild type strain 6471/76 [8]. Phage φR1-37 uses the outer core (OC) hexasaccharide of LPS as a binding receptor [9,10,11,12,13,14]. In addition to serotype O:3, φR1-37 demonstrates a relatively broad host range as it also infects other *Y. enterocolitica* strains representing serotypes O:5,27, O:9 and O:50, as well as *Y. similis* serotype O:9 strains [10]. These serotypes have different surface receptors, yet φR1-37 is able to recognize and infect each of them very specifically [15,16].

The morphology of φR1-37 reveals an icosahedral head of 138 nm in diameter, a short neck, a long contractile tail of 383 nm, and 75 nm tail fibers [10,13]. The genome of φR1-37 is 262,391 bp in size; φR1-37 is considered a jumbo phage based on its structural and genomic analysis [13]. The phage genome is predicted to encode 367 proteins, of which 140 have bioinformatically predicted functions or were identified as structural proteins of the phage [13]. Furthermore, φR1-37 has a long contractile tail with several tail fibers extending from the tail tip [13]. The predicted φR1-37 gene product Gp298 has sequence similarity to phage T4 tail fiber protein Gp37 [17]. Next to gene *g298* is located gene *g297* that encodes a homolog to tail fiber assembly chaperone proteins. Based on the bioinformatic analysis, both genes were predicted to be responsible for producing φR1-37 tail fibers [17]. The structural analysis of φR1-37 tail fiber is yet to be carried out; however, close resemblance to other tail fibers from other phages has been observed [13].

Bacteriocins are proteinogenic toxins secreted by a bacterial strain that exert an antimicrobial effect on the growth of other bacterial strains of the same or closely related bacterial species [18,19]. In the course of investigations of *Yersinia* isolates from food samples, *Y. enterocolitica* strain 29930 (serotype O:7.8/biotype 1A) was found to produce the bacteriocin that targets *Y. enterocolitica* serotype O:3, O:5,27 and O:9 strains [20]. The bacteriocin, named as enterocoliticin based on its target spectrum [21], uses, similar to φR1-37, the LPS OC hexasaccharide as the receptor. The biosynthesis of enterocoliticin is induced either with mitomycin C or ultraviolet light treatments, and the enterocoliticin is subsequently released into the environment by an unknown mechanism. Enterocoliticin is more active on exponentially growing bacteria, as in a stationary bacterial culture, a transient resistance is observed, likely due to the blocking of the enterocoliticin receptors by the capsule-like OPS [20].

Electron microscopy has revealed that enterocoliticin is a phage tail-like particle [20]. Similarly, bacteriocins were reported to be produced by *Y. kristensenii*, *Y. frederiksenii*, and *Y. intermedia* strains [22]. The *Y. enterocolitica* 29930 enterocoliticin tail sheath in the relaxed state is 80 nm long with a diameter of 15 nm, and when contracted shortens to 35 nm, and the diameter increases to 20 nm [23]. Spikes that stick out from the base plate of the particle very likely play a role in adsorption to the OC of susceptible bacteria. Once the enterocoliticin binds, the tail sheath contracts, and the tail tube is pushed through the outer membrane, causing the formation of a pore leading to rapid efflux of potassium ions [20,23]. As enterocoliticin is structurally similar to the tails of even-numbered *Escherichia* T phages that have the injection tube surrounded by a helically arranged protein sheath, it is considered to have evolved from a bacteriophage, possibly resulting from successive reduction in a prophage genome in the host genome [21,24].

In this work, we identified, sequenced, and characterized the gene cluster directing the biosynthesis of enterocoliticin. We identified Gp298 as the RBP of phage φR1-37, and a subsequent local sequence similarity between Gp298 and the predicted *orf39* gene product of the enterocoliticin gene cluster strongly implicated the latter to encode the enterocoliticin RBP. Sequence comparison revealed that both RBPs share a highly conserved 80-85 amino acid (aa) region, which is the sole structural explanation for their shared receptor specificity. To investigate this conserved architecture, we employed AlphaFold3-Multimeric prediction to generate high-confidence structural models of the trimeric Gp298 and Orf39 proteins. Leveraging the structural information provided by these models, we then performed rigorous molecular docking using AutoDock Vina to precisely map the interaction between the Orf39 RBP (the 85-aa motif) and the modeled OC pentasaccharide ligand. By superposing the resulting docked complex onto the Gp298 model, we were able to propose the specific residues responsible for receptor binding in both of the proteins. Collectively, our findings confirm that this 80–85 aa motif constitutes a specialized and conserved carbohydrate-binding structure present in diverse phage tail proteins.

## 2. Materials and Methods

### 2.1. Bacterial Strains, Plasmids and Phages

*Y. enterocolitica* and *Escherichia coli* strains, plasmids and phages used in this work are described in Table 1. Bacteria were grown in lysogeny broth (LB, 10.0 g/L tryptone, 5.0 g/L yeast extract, 10.0 g/L NaCl) or on LA plates (LB supplemented with 1.5% bacto agar). Soft agar was LB supplemented with 0.3–0.7% agar. Antibiotics were supplemented to the media when appropriate as follows: Ampicillin (Amp) at 100 µg/mL, chloramphenicol (Clm) at 12.5 µg/mL, and kanamycin (Kan) at 100 µg/mL. The *Yersinia* strains were grown on LA plates for 48 h at room temperature (RT, about 22 °C), or shaken in 1.3 mL LB for 3–4 h at RT. For enterocoliticin expression, the bacterial strains were grown in LB broth at 20 °C in a shaking incubator at 150 rpm to an OD_588_ of 0.2–0.3. The cultures were then induced with mitomycin C at 1 µg/mL, and incubation was continued at 20 °C at 150 rpm overnight.

### 2.2. Recombinant DNA Methods

Bacterial genomic DNA isolation was carried out with the RTP^®^ Bacteria DNA Mini Kit (Invitek, Berlin, Germany), and plasmid isolation with the GeneJet^TM^ plasmid Miniprep Kit according to Fermentas (St. Leon-Rot, Germany). For polymerase chain reaction (PCR) screening, plasmid DNA was released from bacteria using the boiling method [27]. To obtain high-quality DNA, the plasmid DNA was isolated with the Plasmid Midi Kit (Qiagen, Hilden, Germany) according to the manufacturer’s instructions. Restriction enzyme digestions, ligations and transformations were carried out according to the manufacturer’s recommendations.

PCR was carried out with standard PCR conditions in a total volume of 25 µL. The reaction contained in final concentrations 1×PCR buffer, 0.24 µM each dNTP, 1 mM MgCl_2_, primers at 0.5 pmol/µL, Taq-polymerase (Fermentas GmbH, St. Leon-Rot, Germany) 0.06 U/µL, and 1 ng/µL template DNA. The reaction started with a 3 min heating at 95 °C, followed by 30–35 cycles of 30 s at 95 °C, annealing 30 s at 52–62 °C (temperature depending on primers), and elongation for 0.5–3 min at 72 °C (the time depending on the product size). Final elongation of 5 min at 72 °C was followed by a final cooling at 4 °C. The PCR products used for further experiments were purified with MSB^®^ Spin PCRapace columns from Invitek (Berlin, Germany).

### 2.3. Construction of a Cosmid Library of Y. enterocolitica 29930

A cosmid library was constructed using the SuperCos1 Cosmid Vector Kit following the recommendations of the manufacturer (Stratagene, La Jolla, CA, USA). SuperCos1 carries the Tn*5* kanamycin-resistance (KmR) gene and an ampicillin resistance gene. The cosmid vector was digested with XbaI, dephosphorylated with alkaline phosphatase (Fermentas GmbH, St. Leon-Rot, Germany) and digested with BamHI. Genomic DNA of *Y. enterocolitica* 29930 was prepared using the cetyltrimethylammonium bromide method of DNA extraction [28] and was partially digested with Sau3A (Bsp143I) and ligated with SuperCos1. Using a lambda packaging mix (Gigapack III Gold, Stratagene), the ligation mixture was introduced into *E. coli* VCS257 cells, which were plated on LB agar containing 100 µg/mL ampicillin [29]. The cosmid library will contain the complete genome of a bacterium in overlapping fragments of approximately 30 kb to 46 kb in size when the λ phage packaging system is used.

### 2.4. Plasmid Constructs for φR1-37 RBP Expression

The plasmid constructs for protein expression and purification experiments were synthesized commercially on the plasmid vector pCDF duet-1^TM,^ following the methodology described elsewhere [17,30]. Two different plasmid constructs were generated (Table 1). Plasmid pCP-1 has the φR1-37 gene *g298* inserted at the multicloning site (MCS) 1 of pCDF duet-1^TM^. Plasmid pCP-2 is a derivative of pCP-1 with the φR1-37 gene *g297* inserted at MCS-2. Since the plasmid contributes a start codon and a 6×His-tag at the *N*-terminus of each MCS, gene fragments were synthesized with BamHI/SalI sites added for cloning into MCS-1 to be in frame with the 6×His-tag, and XpnI/XhoI cloning sites were added to clone it into MCS-2. All plasmid constructs were verified by sequencing.

### 2.5. DNA Sequencing and Sequence Analysis

Both Sanger sequencing using primer walking and Illumina high-throughput sequencing methods were used to determine the sequence of Cos141 [31,32]. Sanger sequencing reactions of Cos141 were performed commercially at Eurofins Genomics GmbH (Ebersberg, Germany). Assembly of the contigs was done using Lasergene (SeqMan), DNASTAR Inc., Madison, WI, USA. The Accelrys Software version 2.5 (Accelrys, San Diego, CA, USA). was used for primer design; primers were synthesized by Metabion (Planegg, Germany). The bioinformatic analysis of the Cos141 sequence was carried out with the Genome Annotation Service of the Bacterial and Viral Bioinformatics Resource Center (BV-BRC). The rapid annotations using the subsystems technology tool kit (RASTtk) [33] for bacteria was used for annotation (https://www.bv-brc.org/app/Annotation, accessed 27 March 2025). The annotation required for depositing the sequence at Genbank (accession PV390083) was done by using BLASTP (https://blast.ncbi.nlm.nih.gov/, accessed 27 March 2025) for each potential coding sequence.

The whole genome sequencing (WGS) of *Y. enterocolitica* 29930 was performed as described [31]. The WGS sequence data (raw sequences and assemblies) were deposited at NCBI with accession codes BioProject ID PRJNA1241681 and BioSample ID SAMN47560904. Annotation was performed with the NCBI Prokaryotic Genome Annotation Pipeline (PGAP).

### 2.6. Ultracentrifugation and Ultrafiltration of Enterocoliticin

The release of enterocoliticin from *Y. enterocolitica* 29930 into the culture was induced by the addition of mitomycin C (final concentration of 1 µg/mL, Sigma-Aldrich, Munich, Germany), and isolated as described [20]. Briefly, the bacterial culture was centrifuged at 10,000× *g* for 30 min at 4 °C, and the supernatant was passed through a 0.2 µm filter. The filtrate was pelleted at 230,000× *g* for 2 h. The pellets were resuspended in water and applied to a CsCl step gradient (1.3 to 1.7 g/mL) and centrifuged at 28,000 rpm (141,000× *g*) at 10 °C for 16 h. The band containing enterocoliticin was collected by puncturing the tube with a needle and dialyzed against water. A rapid filtration of the enterocoliticin samples after the CsCl density gradient was also carried out using “Amicon^®^ Ultra-0.5 Centrifugal Filter Devices” from Millipore (Schwalbach, Germany). The isolation procedure of enterocoliticin from the *E. coli* VCS257/Cos141 bacteria was identical.

### 2.7. Transmission Electron Microscopy (TEM)

The CsCl-purified enterocoliticin samples were applied to 400-mesh copper grids (Plano GmbH, Wetzlar, Germany). The grids were fixed with 2.5% glutaraldehyde solution for 1 min and stained with 2% aqueous uranyl acetate solution for 1 min, studied by TEM using a JEM-1010 (JEOL, Tokyo, Japan) at 80 kV accelerated voltage.

### 2.8. MALDI-TOF TOF-MS/MS Analysis of Enterocoliticin Proteins

CsCl-ultracentrifugation-purified enterocoliticin particles were incubated in 10 mM dithiotreitol at 95 °C to reduce disulfide bridges and then alkylated with 50 mM iodoacetamide for 45 min in the dark at RT. Samples were then mixed with SDS-PAGE sample buffer and incubated at 95 °C for 5 min. Protein samples (20–35 µg) were separated in 12.5% and 20% SDS-PAGE, run at 200 V. After the run, the gels were washed with ddH_2_O and stained using BioSafe Coomassie stain (Bio-Rad Laboratories, Hercules, CA, USA). The protein bands were excised (Supplementary Figure S1), cut into smaller pieces and decolorized by repeated washing with 20 mM NH_4_HCO_3_/acetonitrile (50:50 *v*/*v*). The gel pieces were dehydrated with 100% acetonitrile, dried and digested with trypsin. The digestion buffer consisted of 25 mM ammonium bicarbonate (25 µL), 10% acetonitrile (100 µL), 5 mM CaCl_2_ (5 µL) and ultrapure water (870 µL). To 600 µL of this buffer, 15 µL trypsin solution (25 µg in 50 µL 10 mM HCl) was added. After rehydrating and washing the gel slices with trypsin buffer on ice, the gel pieces were covered with a small aliquot of trypsin buffer. Digestion was performed for 16 h at 37 °C. The peptides were extracted from gel slices with 50% acetonitrile/5% trifluoroacetic acid, dried and reconstituted with 0.3% TFA in 60% acetonitrile, and 1 μL sample was transferred onto a stainless-steel target and mixed with 1 µL matrix solution containing 10 mg/mL α-cyano-4-hydroxycinnamic acid in acetonitrile, ddH_2_O, and trifluoroacetic acid (50:47.5:2.5, *v*/*v*).

The MALDI-TOF/TOF MS/MS analysis was performed on an Ultraflex II TOF/TOF instrument (Bruker Daltonik, Bremen, Germany) operated in the reflector mode for MALDI-TOF peptide mass fingerprint (PMF) or lift mode for MALDI-TOF/TOF analysis. PMF and lift spectra were interpreted with the Mascot software version 2.2 (Matrix Science Ltd., London, UK). Database (NCBInr) searches using Mascot were performed via BioTools 3.0 software (Bruker Daltonik) using combined PMF and MS/MS (peptide fragment fingerprinting [PFF]) data sets. The peptide sequences obtained were localized in the annotated Cos141 sequence using WU-blast 2.0 (At present available as AB-BLAST, https://blast.advbiocomp.com/). 

### 2.9. Enterocoliticin Activity Determination

The biological activity of enterocoliticin secreted by *Y. enterocolitica* 29930 or the cosmid-carrying *E. coli* VCS 257 strains was tested as described [34]. The activity was tested on the sensitive *Y. enterocolitica* O:3 strains 13030 and 13169 and on the non-sensitive O:8 strain 8081 and O:5 strain 29807. 20 µL aliquots of enterocoliticin samples were spotted on double-layer agar plates with the test bacteria embedded in the overlay agar. Sensitivity of bacterial strains to enterocoliticin is visible as an area of growth inhibition at the spot. Using serially diluted enterocoliticin samples, the highest dilution showing growth inhibition was determined as the relative activity of enterocoliticin and expressed in activity units (AU per mL) [20].

### 2.10. Phage Microbiology Methods

#### 2.10.1. Preparation of Phage Stocks

Phage φR1-37 was propagated either as described [10,13] or using the semi-confluent plate double-layer agar overlay method [35]. Phages were extracted from soft agar by incubating the plates overlayed with SM buffer (100 mM NaCl, 10 mM MgSO_4_, 50 mM Tris-HCl, pH 7.5, 0.01% *w*/*v* gelation). The soft agar layer with the SM buffer was collected and treated with 200 µL of chloroform for 15 min. The lysate was centrifuged at 5000 rpm, and the supernatant was passed through a 0.2 µm filter. For long-term storage, sucrose was added to the filtered lysate at a final concentration of 8%.

#### 2.10.2. Double-Layer Overlay and Drop Tests

*Y. enterocolitica* strains were grown in 1.3 mL of LB overnight at RT, and the final OD_600_ was determined. The volume of this culture to be added to 3 mL of melted LB soft agar, kept at 55 °C, was calculated using equation 90 µL/OD_600_. Tenfold dilutions of the phage stock were prepared, and 50 µL of each was added to soft agar tubes that were thoroughly mixed and spread evenly on LA plates. The plates were left for 30 min for the soft agar to harden and then incubated at the appropriate growth temperature. The number of plaques was counted to determine the number of plaque-forming units (PFU/mL) of the phage stock [35] using the formula:

*PFU*/mL = *number of plaques* × *dilution factor* × 20.

In the drop test, the molten soft agar (55 °C) was inoculated with the bacterial culture as above and overlaid directly onto the LA plate. After solidification, 10 µL of the phage serial dilutions were spotted onto the bacterial lawn. Plates were incubated overnight at the optimal growth temperature for the host bacteria. The PFU/mL of the phage stock was calculated by counting discrete plaques at an appropriate dilution [36]:

*PFU*/mL = *number of plaques* × *dilution factor* × 100

#### 2.10.3. Efficiency of Plating (EOP)

The efficiency of plating for a given phage was quantified by determining the PFU of alternative bacterial hosts relative to the PFU from its preferred host. The EOP was calculated using the formula:

*EOP* = *PFU*
*in other hosts*/*PFU in its preferred hosts*

#### 2.10.4. Phage Adsorption Assays

Phage adsorption rates were measured by quantifying the reduction in free phage particles in the supernatant after incubation of the phage with the target bacteria. The target bacterial cells were cultured to an OD_600_ of 1.0. Equal volumes (50 µL) of bacterial culture and phage φR1-37 suspension of known titer were combined and incubated for 10 min at RT. The resulting mixture (100 µL) was centrifuged at 5000 rpm for 10 min. The supernatant (50 µL) was then subjected to plaque assay using the double-layer agar method with *Y. enterocolitica* strain YeO3-R1 as the indicator for residual infectious phage particles.

### 2.11. LPS Isolation and Analysis

The LPS of different bacterial strains was isolated using the hot phenol-water extraction, analyzed using deoxycholate-polyacrylamide gel electrophoresis (DOC-PAGE), and silver-stained as described earlier [37].

### 2.12. Protein Expression in E. coli

For the production of recombinant tail fiber proteins, the expression vector-carrying strains were inoculated into 50 mL 2×YT medium (16 g/L tryptone, 10 g/L yeast extract, 5.0 g/L NaCl, pH 6.5–7.5) supplemented with appropriate antibiotics. The cultures were grown overnight at 37 °C on a shaker incubator at 200 rpm. The 50 mL culture was then transferred into 1 L of LB (supplemented with appropriate antibiotic) and allowed to reach the OD_600_ of 0.8. To this culture, isopropyl β-d-1-thiogalactopyranoside (IPTG) was added to the final concentration of 1 mM, and the induced culture was grown overnight at 30 °C on a shaker at 200 rpm. The production was monitored by SDS-PAGE analysis with 12% gel stained with InstantBlue (Expedon) for 5 min and visualized with a Gel DOC™ XR+ imaging system (Bio-Rad Laboratories, Hercules, CA, USA).

For immunoblotting, the proteins were transferred from the gel to a Whatman Protran nitrocellulose membrane, pore size 0.45 µm (Merck KGaA, Darmstadt, Germany) using a Thermo Scientific^TM^ Owl^TM^ semi-dry-electroblotting-system (Thermo Fisher Scientific, Waltham, MA, USA). The membrane was blocked with 5% *w*/*v* non-fat dry milk in TBST buffer (50 mM Tris-HCl, 0.05% Tween 20, 150 mM NaCl pH 7.6) for 1 h at RT. Following TBST washes, the membrane was incubated with the primary anti-His-tag antibody [1:10,000] in TBST overnight at 4 °C on a shaker. After three TBST washes, the membrane was incubated with the horse-radish peroxidase conjugated secondary anti-mouse IgG polyclonal antibody (1:2000 in 5% non-fat dry milk in blocking buffer) for 1 h at RT. Subsequently, after additional TBST washes, the proteins were visualized using enhanced chemiluminescence (ECL) solution (0.1 M Tris-HCl pH 8.5, 5.3 mM hydrogen peroxide, 1.25 mM luminol, 0.2 mM coumaric acid). The membrane was finally exposed to a light-sensitive film (Kodak, Rochester, NY, USA) in a dark room.

Bacterial cells were harvested after induction by centrifugation (3000× *g* for 20 min at 4 °C) and resuspended in 20 mL of Buffer A (50 mM sodium phosphate buffer, 300 mM NaCl, 20 mM imidazole, pH 8.0) supplemented with lysozyme (5 mg/mL) and protease inhibitor cocktail (Roche, Basel, Switzerland). After 30 min incubation on ice, the cells were lysed by sonication (six 1 min cycles). The lysate was clarified by centrifugation (10,000× *g* for 20 min) and the clear supernatant was collected. The supernatant was incubated with Ni-NTA resin overnight at 4 °C with constant agitation. The resin was collected by low-speed centrifugation (120× *g*, 3 min) and washed 5 times with 40 mL of buffer A. The His-tagged proteins were eluted by Buffer B (50 mM sodium phosphate buffer, 300 mM NaCl, 500 mM imidazole, pH 8.0). Buffer exchange was performed using a PD10 desalting column. The column was equilibrated with 20 mL of phosphate buffered saline (PBS, pH 7.4). The protein sample was loaded to the column and the eluted fractions were collected and stored at 4 °C. The protein concentration was determined on spectrophotometer at OD_260_. The purity of the eluted proteins was assessed by SDS-PAGE gel electrophoresis.

Native polyacrylamide gel electrophoresis (Native-PAGE) was performed to analyse the conformational state of purified proteins under non-denaturing conditions. For this purpose, 5% stacking gel (30% Acrylamide/Bis 0.67 mL; 0.375 M Tris pH 8.8, 4.275 mL; 10% ammonium persulphate 50 μL; tetramethylethylenediamine [TEMED] 5 μL) and 10% resolving gel (30% Acrylamide/Bis 3.4 mL; 0.375 M Tris pH 8.8 6.49 mL; 10% ammonium persulphate 100 μL; TEMED 10 μL) was prepared. Protein samples were mixed with an equal volume of 2× sample buffer (62.5 mM Tris-HCl, pH 6.8, 25% glycerol, 1% bromophenol blue) and 10 µL of the mixture was loaded onto the native polyacrylamide gel. Electrophoresis was performed in running buffer (25 mM Tris 192 mM glycine) at 80 V and then stained with InstantBlue (Expedon) for 5 min. Gel images were captured using a Gel DOC™ XR+ imaging system (Bio-Rad Laboratories, Inc.).

### 2.13. High Pressure Liquid Chromatography (HPLC)

High-resolution preparative gel filtration chromatography was performed using a HiLoad 16/60 (GE Healthcare, Little Chalfont, UK) column. The chromatographic system was primed to remove all the air bubbles before vertical installation of the column. The column was equilibrated with two column volumes of 80% EtOH followed by two column volumes of Pure Aqua. Protein samples were loaded onto the column using a Hamilton syringe. Proteins were eluted with PBS. Elution was monitored by UV absorbance at 280 nm, and protein fractions were collected and stored at −20 °C.

### 2.14. Interaction of Dynabeads-Immobilized LPS with Tail Fiber Protein

Five mg of Dynabeads (M-280 Tosylactivated, ThermoFisher Scientific, Waltham, MA, USA) were coupled with 300 µg of *Y. enterocolitica* O:3 LPS following the manufacturer’s protocol. To evaluate phage binding affinity, the LPS-conjugated Dynabeads were incubated with phage φR1-37, and subsequent adsorption was quantified using a phage adsorption assay. For tail fiber protein binding assessment, the LPS-conjugated Dynabeads were separately exposed to purified Gp298 protein. The bead-protein complexes were subsequently analysed by SDS-PAGE to confirm the molecular interaction between the tail fiber protein and the immobilized O:3 LPS.

### 2.15. Blocking of Host Cell Surface with Purified Tail Fiber Protein

Bacterial host cells were cultured to OD_600_ of 1.0, after which 10 µL aliquots were harvested and washed twice with 1 mL PBS. For the experimental group, purified tail fiber protein (5 µL at 0.7 µg/µL) was added to the washed bacterial pellets, while control samples received no protein treatment. All samples were brought to a final volume of 100 µL with PBS. The experimental samples containing tail fiber protein were incubated at 4 °C for 4 h, followed by the addition of φR1-37 phages to both experimental and control samples. After a 25-min incubation period, all samples were subjected to two additional washing steps with 1 mL PBS and subsequently resuspended in 100 µL PBS. The number of infected bacteria for both protein-treated and untreated host cells was determined using the standard double-layer agar overlay technique.

### 2.16. Modeling of Protein Multimers

#### 2.16.1. Ligand Structure Preparation

Initial oligosaccharide structures were constructed using the CarbBuilder software, version 2.1.45 [38]. These models were subsequently refined and energy-minimized in Avogadro (version 1.2.0) [39], utilizing both steepest descent and conjugate gradient methods to achieve a low-energy three-dimensional conformation.

#### 2.16.2. Protein Multimer Prediction

The initial trimeric structures for the receptor binding proteins (RBPs), Orf39 (Protein_id YBJ30793.1) and Gp298 (UniprotKB ID: G4KKN6), were predicted independently. We used the multimer prediction functionality of the official AlphaFold Server (https://alphafoldserver.com/), powered by the AlphaFold3 [40] deep learning model. For each RBP, the sequence was submitted, and the number of chains was specified as three using default server settings, yielding five structural models per protein.

#### 2.16.3. Quality Assessment and Data Extraction

Model quality was assessed primarily using the predicted Local Distance Difference Test (pLDDT) metric (range 0–100), which quantifies the confidence in local residue positioning. Models with pLDDT values above 90 indicated very high local confidence, while scores between 70 and 90 suggested generally reliable structural regions. For both proteins, the model with the highest overall average pLDDT score was selected for further complex analysis.

Crucially, the raw structural analysis data, including the Predicted Aligned Error (PAE) matrix—which is essential for assessing the confidence of domain and inter-chain orientations—was not included in the standard downloadable package. Therefore, we programmatically extracted the raw PAE data from the server’s Application Programming Interface (API) during the run visualization phase using web developer tools. This PAE data was plotted to confirm confidence in the relative orientations of domains and chains (i.e., inter-chain and intra-chain confidence) within the trimeric models. All predicted 3D structures of trimeric Orf39 and Gp298 were visualized and analyzed using the molecular visualization software PyMOL^TM^ Molecular Graphics System Version 3.1.0 (Schrödinger Inc., New York, NY, USA).

### 2.17. AlphaFold2-Driven Modeling and Subsequent Molecular Docking Analysis

#### 2.17.1. AlphaFold2-Based Modeling of Orf39

Due to external licensing constraints governing the use of AlphaFold3 models in quantitative molecular docking analyses, the rigid receptor structure for the docking experiments was generated using AlphaFold2-Multimer (version 2.3.1) [41]. This was implemented via the high-throughput interface ColabFold (version 1.5.5) [42], specifically utilizing the alphafold2_multimer_v3 model type.

To enforce the required trimeric stoichiometry, the Orf39 RBP monomer sequence (corresponding to the specific 85-amino acid binding domain) was concatenated three times, separated by a 120-residue flexible linker. Multiple Sequence Alignment (MSA) was generated using the sensitive MMseqs2 server [43] by querying the UniRef90 [44] and Mgnify [45] protein sequence databases. The resulting AlphaFold2 trimer model with the highest Interface Predicted Template Modeling (ipTM) score was selected for subsequent docking.

The predicted fold of the full-length Orf39 trimer generated by AlphaFold2 was found to be highly congruent with the corresponding AlphaFold3-Multimer prediction (see Section 2.16). This strong structural concordance validated the quality of the AlphaFold2 multimeric model. The specific 85-amino acid Receptor Binding Domain (RBD) motif was subsequently extracted from this selected AlphaFold2 full-length trimer and designated as the rigid receptor for the molecular docking exercise.

#### 2.17.2. AutoDock Vina Docking Protocol

Molecular docking was conducted using AutoDock Vina (version 1.2.3) [46,47] to computationally model the interaction between the extracted Orf39 RBD trimer (the rigid receptor) and the prepared, energy-minimized pentasaccharide ligand.

The initial conversion of both the receptor and ligand files into the required AutoDock PDBQT format was performed using the MGLTools suite (version 1.5.7) [48] and Open Babel (version 2.4.1) [49]. To ensure full compliance with the Vina atom-typing standard and the accurate calculation of torsional degrees of freedom, an in-house Python script was subsequently employed to rigorously clean up the ligand’s PDBQT file.

A blind docking strategy was employed using a large cubic grid box to ensure comprehensive searching of the Orf39_RBP’s surface. The box was centered at Center Coordinates: X = 21.17, Y = 36.612, and Z = 0.168 with Dimensions: 60.0 × 30.0 × 30.0 Å^3^. Vina parameters were set to an exhaustiveness of 32, reporting a maximum of 18 output binding modes within an energy range of 5.0 kcal/mol of the best pose.

#### 2.17.3. Pose Selection and Interaction Profiling

The generated binding poses were visually inspected and ranked by affinity (see Table S1 for the details of the 18 AutoDock Vina poses). The mode 2 (binding affinity ΔG = −6.273 kcal/mol) was chosen as the final representative pose over the highest-affinity mode (Mode 1). Mode 2 was selected because it localized specifically to a clear inter-subunit binding cleft and supported the trimeric folding of Orf39. Detailed non-covalent interactions for this selected pose were characterized using the Protein-Ligand Interaction Profiler (PLIP) (version 2.4.0) [50].

## 3. Results

### 3.1. Identification and Cloning of the Enterocoliticin Biosynthesis Genes of Y. enterocolitica 29930

The enterocoliticin biosynthesis gene cluster was identified using the following strategy. The N-terminal aa sequences of the enterocoliticin particle proteins were determined and a BLASTP search with two of them on the *Y. pestis* strain CO92 proteome identified two putative phage-related proteins annotated as a phage tail sheath protein (WP_002208856.1) and a phage tail tube protein (WP_002208855.1). PCR primers were designed based on the genes coding for the proteins and used to screen the cosmid library of *Y. enterocolitica* 29930. The primer pair, tailshif3 and tailshif4 (Table S2), was used to screen the *Y. enterocolitica* strain 29930 cosmid library as described below.

#### 3.1.1. Identification of the Cosmid Carrying the Enterocoliticin Gene Cluster

Cosmids carrying the enterocoliticin biosynthesis gene cluster were identified by PCR screening the *Y. enterocolitica* 29930 cosmid library in *E. coli* VCS257. Close to 500 cosmid clones were screened using the primer pair tailshif3 and tailshif4. A few cosmid clones yielded a PCR product of the correct size. The supernatants of all the PCR-positive cosmid clones were tested for enterocoliticin production, and two clones showed inhibitory activity against enterocoliticin-sensitive strains. The cosmid clone with the higher enterocoliticin activity (termed Cos141) was chosen for further experiments.

#### 3.1.2. Characterization of Cos141

The culture supernatant of *E. coli* VCS257/Cos141 showed inhibitory activity against the susceptible *Y. enterocolitica* O:3 strains. To quantify the inhibitory activity, the supernatant was sterile filtered and serial dilutions were tested on double-layer agar plates containing susceptible and non-susceptible *Y. enterocolitica* strains [20]. To confirm that phage tail particles were responsible for the inhibition, the supernatant was subjected to CsCl-gradient ultracentrifugation, and the material recovered from a band was tested for growth inhibition on *Y. enterocolitica* strains. The ultracentrifugation increased the inhibitory activity on the *Y. enterocolitica* O:3 strains 13169 and DSM 13030 significantly, while no growth inhibition was observed on the non-susceptible *Y. enterocolitica* serotype O:8 and O:5 strains (Table 2).

**Figure 1:**
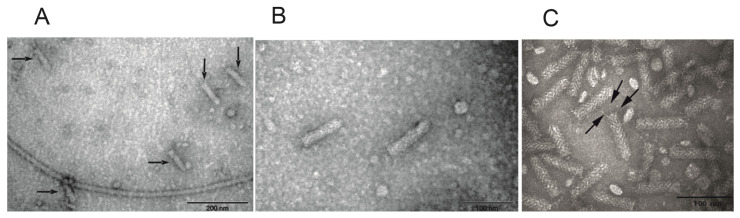
Electron micrographs of enterocoliticin particles from *E. coli* VCS257/Cos141 (**A**,**B**) and from *Y. enterocolitica* 29930 (**C**). The scale bar in (**A**) is 200 nm, and 100 nm in (**B**) and (**C**). The arrows in (**A**) indicate the enterocoliticin particles, and in (**C**), the tail fibers.

The presence of phage tail particles in the ultracentrifuge-purified sample from the culture supernatant of *E. coli* VCS267/Cos141 was investigated using TEM, which showed clearly the presence of phage tail particles whose dimensions corresponded to those of *Y. enterocolitica* 29930 (Figure 1). The amount of the synthesized particles by the *E. coli* VCS267/Cos141 clone was significantly lower than the production of particles by the native producer *Y. enterocolitica* 29930. Under the same cultivation and isolation conditions, enterocoliticin samples derived from strain 29930 possessed activity of 204.800 AU per mL against *Y. enterocolitica* 13196 and 409,600 AU per mL against *Y. enterocolitica* 13030.

#### 3.1.3. Sequence Analysis of Cos141

**Figure 2:**
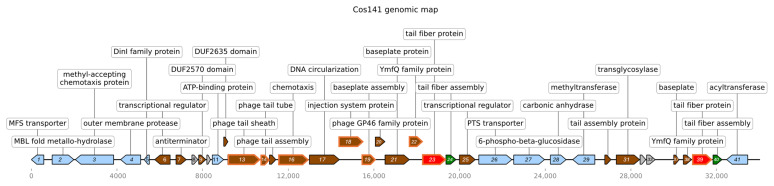
Gene organization of the enterocoliticin gene cluster in Cos141. The arrows represent the predicted genes that are numbered according to the annotation presented in Table S4. The arrowhead indicates the transcription direction. The colors indicate predicted functions of the gene products. Brown, phage-related genes; Light blue, phage-non-related genes; Red, phage tail fiber; Green, Tail fiber assembly. The orange framing indicates genes whose gene products were identified by MALDI-TOF-MS analysis (Table S5). The gene map was generated using the Linear Genome Plot tool [52].

The nucleotide sequence of Cos141 was determined by primer walking combined with PCR amplification. The *Y. enterocolitica* 29930 DNA fragment inserted in Cos141 was 34,018 bp in size (Figure 2). Through a series of PCR amplifications designed to achieve overlapping PCR products, we could show that Cos141 is colinear with the 29930 genome and not composed of several ligated genomic DNA fragments. This was confirmed when the whole genome sequence of strain 29930 was obtained, showing that Cos141 insert is 100% identical to the genome 29930.

The results of the bioinformatic annotation of Cos141 insert sequence are shown in Supplementary Table S4. The Cos141 insert contains altogether 42 predicted genes (*orf1*–*orf42*, Table S4) with two regions of genes that encode potential phage proteins. The first region comprises the genes *orf6* to *orf24*. The second region stretches from *orf30* to *orf40*. The latter gene cluster contains several very short Orfs (<100 aa) of which three gene products are annotated as pseudogenes. However, the first region contains a linear order of genes that encode proteins that are similar to proteins of the *E. coli* phage Mu that build the contractile injection apparatus of the Mu phage [51]. The Orf17 and Orf18 proteins may form the central hub complex (similar to Mup43 and Mup44), whereas the Orf20, Orf21 and Orf22 proteins build the wedge (similar to Mup46, Mup47 and Mup48). Orf19 could be a baseplate spike protein (Mup45) and Orf23 a phage tail fiber protein (Mup49). There are some more gene products functioning as structural components of the injection apparatus (e.g., Orf11 and Orf13 annotated as phage tail sheath proteins, and Orf17 as phage tape measure protein).

Upstream of the genes encoding the contractile injection apparatus are genes that may play a role in the regulation of the biosynthesis. The gene *orf6* product is annotated as phage repressor protein CI, and both the gene *orf7* and *orf9* products are annotated as antitermination proteins Q (Table S3). Enterocoliticin is a tailocin similar to the contractile R-type pyocins of *Pseudomonas* strains. Some regulatory genes of R-type pyocins are also located upstream of the pyocin-encoding genes. Like enterocoliticin, the R-type pyocins lack genes for head assembly and DNA packaging [53,54]. The comparison of the genomes of the tailocins (R-type pyocins and enterocoliticin) to phage Mu reveals that the arrangement of the genes coding for the injection apparatus is similar. In the case of phage Mu, these genes belong to the group of “late” genes, which are translated shortly before virions are released [53].

#### 3.1.4. Mass Spectrometry of Enterocoliticin Proteins

By determining peptide sequences derived from proteins of enterocoliticin particles, information can be gained about their encoding genes. For this purpose, SDS-PAGE separated protein bands of the CsCl gradient-purified enterocoliticin of *Y. enterocolitica* 29930 were analyzed by MALDI TOF after their separation in denaturing SDS-PAGE gels, followed by trypsin digestion. The protein bands isolated from SDS-PAGE gels were numbered and cut out (red boxes in Supplementary Figure S1). The tryptic peptides identified in MS were compared to the translated gene products of Cos141. A striking finding was that some peptides assigned to Orf13 and others to Orf23 were found in protein bands of different sizes. A possible explanation is that due to the particle structure of enterocoliticin, some structural proteins may be post-translationally modified to allow the autoassembly of the tailocin particle, or alternatively, the proteins have in-gel interactions with other proteins that slow down their migration in the gel. The analysis showed that several tryptic peptides match peptide sequences of the Cos141 gene products. All matches between peptides and gene products of Cos141 coding sequences were found in the first cluster of phage genes (*orf6* to *orf24*). Table S5 shows the identified peptides and the corresponding nucleotide positions in the Cos141 sequence. In total, peptides of gene products of Orf11, Orf13, Orf14, Orf16, Orf18, Orf19, Orf22, and Orf23 were detected. As the N-terminal parts of Orf23 and Orf39 are highly similar, the two tryptic peptides identified for Orf23 were also identified for Orf39 (with slightly lower score).

In Figure 2, the genetic map of the Cos141 sequence is shown, and the genes whose gene products were found in the MS analysis are marked with an orange frame. All identified phage particle proteins can be attributed to structural components of the contractile injection apparatus.

### 3.2. Identification of Phage φR1-37 RBP

#### 3.2.1. EOP and Phage Adsorption Kinetics

**Figure 3:**
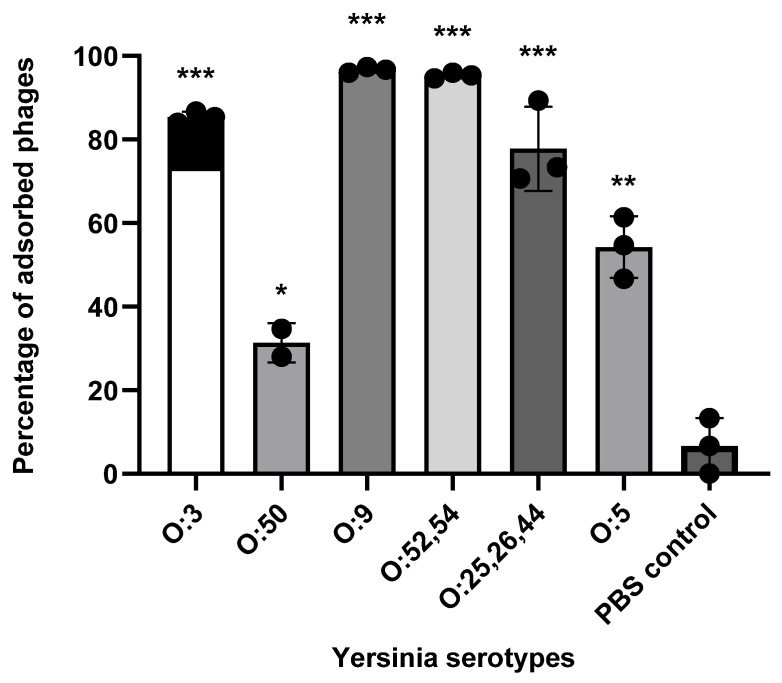
Phage adsorption efficiency across *Yersinia* host strains. Data represent the percentage of phages adsorbed to the bacterial surface after 10 min of incubation. Included are *Y. enterocolitica* strains YeO3-R1 (O:3), 3229 (O:50), 18425/83 (O:25,26,44), and 14779/8 (O:5), *Y. similis* strain R708Ly (O:9), and *Y. intermedia* strain 821/84 (O:52,54). A one-tailed Student’s *t*-test was used to estimate statistical significance compared to the PBS control (* *p* < 0.05, ** *p* < 0.005, *** *p* < 0.0005). Error bars indicate standard deviation (*n* = 3).

Phage φR1-37 stock was prepared to a final concentration of 10^11^ PFU/mL, and its EOP was evaluated on the five susceptible strains and compared to the original host strain *Y. enterocolitica* YeO3-R1 (Table 3). The results show that while the *Y. similis* and *Y. intermedia* strains were infected almost as efficiently as YeO3-R1, the O:50 strain 3229 had a 100-fold lower EOP. In contrast, the *Y. enterocolitica* serotype O:25,26,44 and O:5 strain EOPs were very low. Differences between the strains were also seen in the adsorption assay; the strains with the highest EOPs (serotype O:3, O:9 and O:52,54 strains) also demonstrated the strongest adsorption potential for phage φR1-37, and the strains with lower EOPs (O:5, O:50, and O:25,26,44) showed clearly lower adsorption potential (Figure 3).

#### 3.2.2. Expression, Purification, and Trimerization of Recombinant Gp298

The putative RBP of phage φR1-37 was identified through genomic annotation analysis (GenBank accession number NC_016163.1). The lack of demonstrable similarity between tail fiber proteins of different phages often prevents their direct identification. In the genomes of myoviruses, however, the long tail fiber (LTF) genetic locus is often conserved, which guides the prediction of the gene functions. To determine similarity to described proteins and genome sequences in the NCBI database, analyses were performed using BLAST algorithms (http://www.ncbi.nlm.nih.gov/BLAST/, accessed 27 March 2025) with default parameters [55,56]. HHpred analysis (https://toolkit.tuebingen.mpg.de/tools/hhpred, accessed 27 March 2025) was then used to detect remote protein homology and predict its potential structure [57]. Based on these in silico analyses, the product of *g298* (704 aa) was identified as the probable tail fiber RBP of φR1-37, as it shares similarity to phage T4 tail fibers Gp37 and Gp12 that are involved in host recognition. In addition, inspection of the adjacent gene product Gp297 (228 aa) suggests a chaperone role in tail fiber assembly based on the presence of a Caudovirales Tail Fiber Assembly Protein domain at aa interval 100–222 (pfam02413).

**Figure 4:**
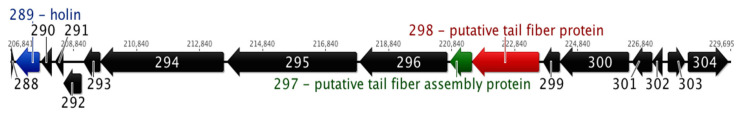
The physical map of the φR1-37 genome region carrying the genes encoding the putative tail fiber protein Gp298 (red) and the putative tail fiber assembly protein Gp297 (green) [13,17]. The arrow-heads indicate the transcription direction.

**Figure 5:**
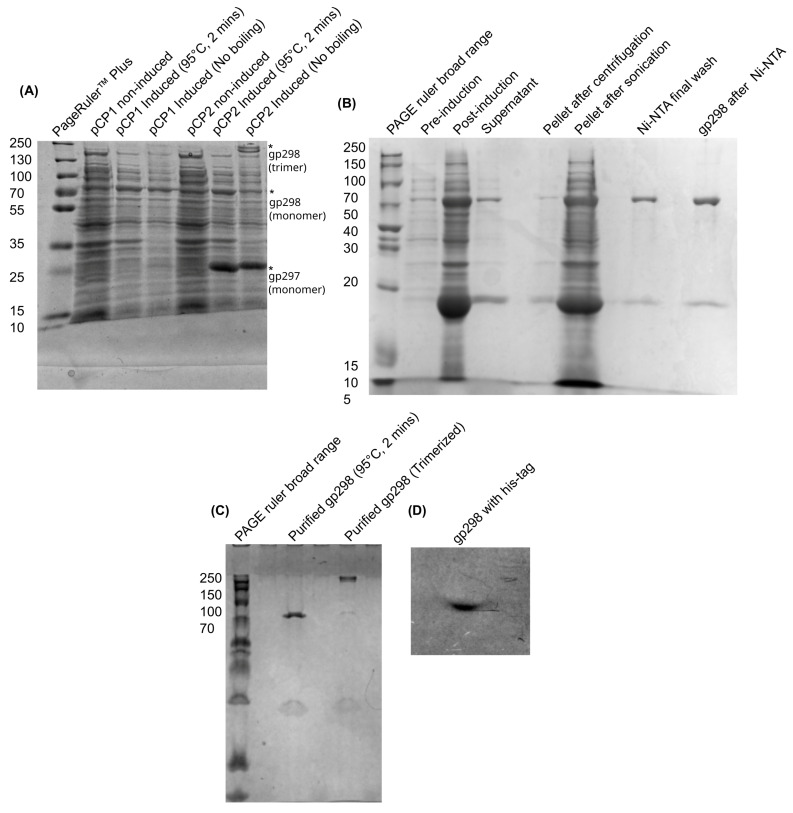
Purification of Gp298. (**A**) SDS-PAGE analysis of overexpression from pCP-1 and pCP-2 plasmids. The non-induced and induced samples were loaded side by side alongside the samples without heat-treatment. The Gp297 and Gp298 bands are indicated by asterisks. (**B**) Large-scale production of pCP-2 plasmid expressing the Gp298 and Gp297 proteins purified by NiNTA beads. (**C**) Gp298 protein analyzed under reducing conditions with and without heat treatment (98 °C, 2 min). (**D**) Western blot picture of purified Gp298 protein.

Figure 4 illustrates the putative tail fiber protein-encoding gene (*g298*) and the adjacent assembly protein-encoding gene (*g297*). Expression constructs pCP-1 and pCP2 (Table 1) were generated by cloning *g298* with or without the putative chaperone gene *g297* into the pCDF-Duet-1™ vector. Protein expression profiles were further characterized and confirmed using SDS-PAGE and ProtParam tool (https://web.expasy.org/protparam/protpar-ref.html, accessed 27 March 2025) [58] indicating that plasmids pCP-1 and pCP-2 overexpressed proteins of the molecular weights consistent with their expected sizes, i.e., 74 kDa for pCP-1, and 74 and 20 kDa for pCP-2 (Figure 5A).

Following purification, it was determined that the products expressed by pCP-1 failed to trimerize into their native conformation (Figure 5A). The plasmid lacked gene *g297* encoding the predicted tail fiber assembly protein that is necessary for the trimerization of the tail fiber protein. Consequently, only pCP-2 was selected for large-scale protein purification and subsequent experimental analyses. The results of the pCP-2 protein purification process are presented in Figure 5B, demonstrating the successful isolation and preparation of the protein for further characterization.

A critical feature of the recombinant tail fiber proteins is their ability to trimerize into their native, functional conformation. To assess this, purified Gp298 obtained from the pCP-2 construct was analyzed for trimerization potential. Protein samples were subjected to SDS-PAGE under non-reducing conditions (i.e., SDS-free sample buffer without boiling prior to loading) to preserve potential higher-order structures. These results were compared with samples of the same protein after boiling in reducing sample buffer, which served as a control to confirm the monomeric state of the protein. This comparative analysis allowed for the evaluation of trimerization capability, a key determinant of the protein’s functional integrity (Figure 5C). The boiled samples of pCP-2 exhibited a molecular weight of 74 kDa, consistent with the expected size of the monomeric form of the protein. In contrast, the unboiled pCP-2 sample displayed a molecular weight of approximately 250 kDa, indicative of a higher-order oligomeric structure, likely a trimer. A faint band at 74 kDa was also observed in the unboiled sample, suggesting the presence of a small fraction of monomeric protein. Additionally, a 27 kDa band corresponding to the chaperone protein was detected in both boiled and unboiled samples, confirming that the tail fiber assembly protein co-purified with the tail fiber protein.

To further validate the identity of the purified protein, Western blot analysis was performed using an anti-His antibody, followed by detection with a horseradish peroxidase-conjugated secondary antibody. Figure 5D presents the X-ray film developed from the Western blot, confirming the presence of the His-tagged tail fiber protein and corroborating the results obtained from SDS-PAGE. These findings collectively demonstrate the successful purification and trimerization of the recombinant Gp298 protein, as well as the co-purification of the essential chaperone protein required for its native assembly.

The protein samples were further purified through HPLC. The HPLC was carried out to separate the chaperone Gp297 from the tail fiber protein Gp298 sample. The purified protein preparation of pCP-2 was subjected to an HPLC run and eluted with PBS. The proteins were eluted as a large peak. Analysis of the recovered fractions in SDS-PAGE showed that HPLC failed to separate Gp298 from its chaperone Gp297, resulting in identical two protein bands as in Figure 5B.

#### 3.2.3. φR1-37 Interacts with *Y. enterocolitica* O:3 via the Gp298 Putative RBP

**Figure 6:**
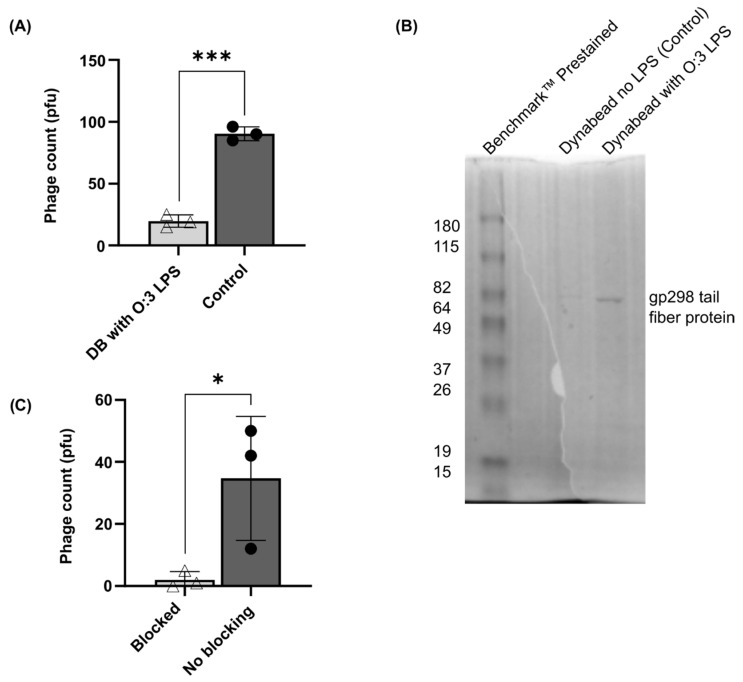
Gp298 interaction with *Yersinia* LPS. (**A**) PFU of φR1-37 bound to DB coupled with *Y. enterocolitica* O:3 LPS. (**B**) SDS-PAGE gel depicting purified Gp298 protein bound to DB coupled with *Y. enterocolitica* O:3 LPS. (**C**) φR1-37 infection of *Y. enterocolitica* O:3 with surface blocked with gp298 protein. Error bars indicate standard deviation (*n* = 3). Significance testing was performed using student’s *t*-test * *p* < 0.05, *** *p* < 0.0005.

LPS from *Y. enterocolitica* O:3 was isolated and resolved using DOC-PAGE gel electrophoresis (Figure S2). Purified O:3 LPS was subsequently coupled to tosylactivated Dynabead M-280 (DB) particles, and phage φR1-37 was assessed for binding affinity to the immobilized LPS. Analysis of the supernatant revealed a significant reduction in free phage particles in the presence of DB-coupled O:3 LPS (Figure 6A), indicating substantial binding of φR1-37 to the O:3 LPS substrate. To further characterize the molecular basis of this interaction, purified tail fiber protein Gp298 was introduced to the DB-O:3 LPS complex. SDS-PAGE analysis (Figure 6B) demonstrated the presence of bound Gp298 protein on the surface of DB particles coupled with O:3 LPS. These findings provide compelling evidence supporting our hypothesis that Gp298 functions as the RBP for φR1-37, and that phage infection is initiated through the specific interaction between the Gp298 tail fiber protein and the O:3 LPS of *Y. enterocolitica*.

A cell surface blocking experiment with purified Gp298 protein was carried out by incubating YeO3-R1 bacteria with 3.5 µg of purified Gp298 and infecting the bacteria with phage φR1-37 (Figure 6C). While the non-treated control bacteria produced an average of 35 plaques, only two were obtained with the blocked bacteria, suggesting that the purified Gp298 effectively blocked the bacterial surface. The difference was statistically significant.

### 3.3. Identification of Enterocoliticin RBP

**Figure 7:**
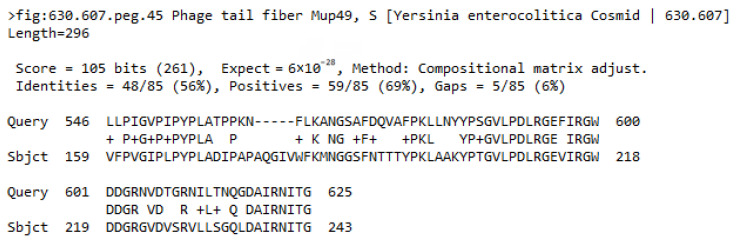
BLASTP alignment when searching Cos141 predicted protein sequences for similarity to Gp298.

Based on the assumption that φR1-37 and enterocoliticin RBPs (that utilize the same receptor to adsorb on *Y. enterocolitica* O:3 bacteria) would demonstrate sequence homology, the nucleotide sequences of φR1-37 and the insert in Cos141 were searched for similarity, which, however, was not found. In addition, Dotplot comparison of the sequences showed that very short non-significant identity stretches were randomly scattered all over the plot. To narrow down the search, the gene *g298* sequence was aligned against the Cos141 insert with similar results. As a last resort, the aa sequence of Gp298 was used to search all the predicted gene product sequences of Cos141. The only significant hit was to Orf39, annotated promisingly as phage tail fiber protein (Figure 7), thus suggesting that this 296-aa protein would be the enterocoliticin RBP, and that the ca. 80–85 aa regions of the two proteins constitute the RBDs of the proteins.

### 3.4. Evolutionary Considerations

The Gp298, Orf39 and the ca. 80 putative binding site aa sequences were used to search the sequence databases by BLASTP. All the hits to Gp298 were due to the 80 aa binding site (Figure S3). On the contrary, identical proteins to enterocoliticin Orf39 were abundant in the databases (Figure S4), with most hits on the N-terminal 150-aa region. A subpopulation of hits included partial similarity to the 85 aa binding site or to the full-length protein. Significantly, all 100 Orf39 and its 85 aa hits were present in the *Yersinia* genomes except for three *Pectobacterium* hits for the latter (Figure S5). Closer inspection of the hits revealed that they were encoded by genes located both in complete prophages of ca 51 kb in size or in truncated ones of ca 15 kb in size. On the contrary, the Gp298 80 aa binding site hits had a very wide diversity (Figure S6), representing phage tail proteins from *Escherichia*, *Dickeya*, *Cronobacter*, *Salmonella*, *Citrobacter*, *Enterobacter*, *Pseudomonas*, *Y. pseudotuberculosis*, *Rahnella*, *Leminorella*, *Pectobacterium*, and *Ewingella*. Figure S7 illustrates the alignment of the top 100 BLASTp hits of both binding site sequences, demonstrating the overall conservation of the identified carbohydrate-binding motif.

### 3.5. Computational Structural Analysis of RBPs and Receptor-Binding

To understand and visualize the structural basis for the shared receptor-binding of the Orf39 and Gp298 tail fiber proteins, we modeled their 3D-structures both in the native trimeric form and in the presence of a putative oligosaccharide (see below). This approach was essential for identifying the putative receptor-binding site and providing a physical explanation for their functional homology. All models were assessed for quality using the pLDDT scores, which are overlaid on the ribbon diagrams of the protein models (Figure S8). The PAE plots per residue of the models, along with their pTM and ipTM values, were also taken into consideration when the structures were completed.

#### 3.5.1. The OC Oligosaccharide Model

**Figure 8:**
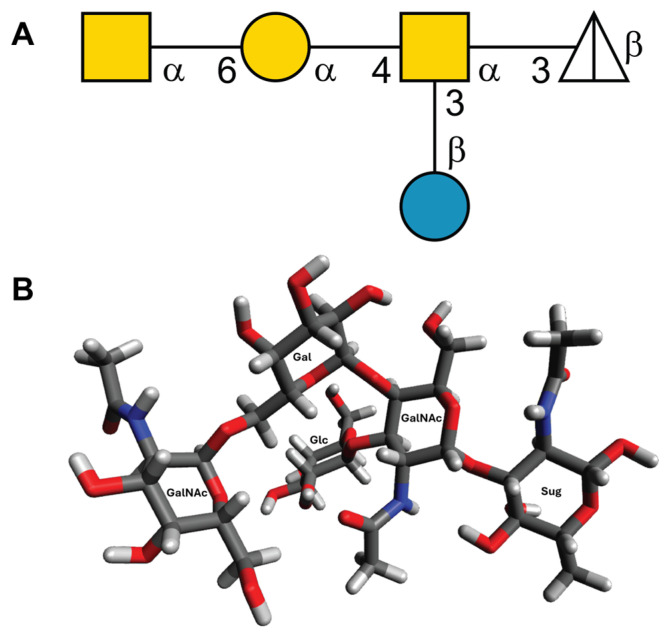
OC pentasaccharide structure. (**A**) Schematic representation in SNFG format of a pentasaccharide structural element from the LPS OC region of *Y. enterocolitica* serotype O:3. The sugar residues in SNFG format [59] were drawn by GlycanBuilder [60] and are denoted as follows: 2-acetamido-2,6-dideoxy-d-*xylo*-hex-4-ulopyranose (Sug*p*) is represented by a triangle filled with white color and divided by a bisector, *N*-acetyl-d-galactosamine (Gal*p*NAc) by a square filled with yellow color, d-galactose (Gal*p*) by a circle filled with yellow color and d-glucose (Glc*p*) by a circle filled with blue color. The OC region contains an additional Glc*p* residue β-(1⟶6)-linked to the terminal Gal*p*NAc residue, and a Sug*p* residue substitutes a heptose residue in the inner core region [12]. (**B**) 3D Molecular model of the pentasaccharide built using CarbBuilder [38] and further modified in Avogadro (version 1.2.0) [39]. Carbon atoms are represented in dark grey, oxygens in red, nitrogens in blue and hydrogens in light grey. At the glycosidic torsion angles of the (1⟶3)- and (1⟶4)-linked sugar residues the conformation is referred to as *syn* where the hydrogen atoms at the anomeric carbon atoms and the glycosyloxylated carbons are in close proximity; at the α-d-Gal*p*NAc-(1⟶6)-linked sugar residue the torsion angle *ψ* (C1′-O6-C6-C5) has an antiperiplanar conformation and the torsion angle *ω* (O5-C5-C6-O6) has the *tg* conformation in the penultimate sugar residue (galactose).

The 3D-structure of the pentasaccharide moiety of the OC hexasaccharide, representing the minimal structure recognized by enterocoliticin [11], was modeled (Figure 8) using CarbBuilder [38]. The resulting structure was then subjected to steepest descent and conjugate gradient energy minimizations in Avogadro [39] achieve a low-energy conformation for subsequent prediction of molecular structures of glycan-protein complexes. The X, Y, and Z dimensions of the model are 11.60, 11.31, and 17.88 Å, respectively.

#### 3.5.2. Predicted Trimeric Structures of Orf39 and Gp298

**Figure 9:**
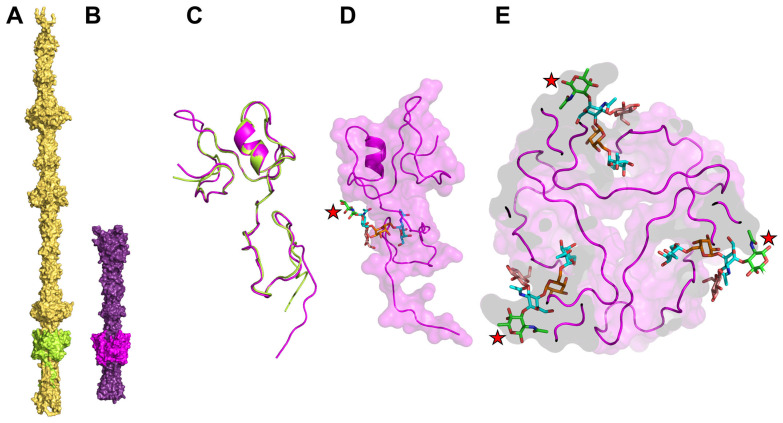
Structural modeling of the trimeric tail fiber proteins and the prediction of the receptor binding domain. (**A**). Gp298 trimer model (yellow, length 494 Å, diameter 40–55 Å); N-terminus at the top. The predicted RBD is indicated in lime. (**B**). Orf39 trimer model (purple, length 213 Å, diameter 39–45 Å); N-terminus at the top. The predicted RBD is indicated in magenta. (**C**). Structural alignment of the RBDs of Gp298 (lime) and Orf39 (magenta). (**D**). Docked pentasaccharide with Orf39 RBD monomer model with the sugar residues differently colored (Sug*p*, green; Gal*p*NAc, turquoise; Gal*p*, orange; Glc*p*, light brown). (**E**). Enterocoliticin Orf39 trimer RBD model in complex with the pentasaccharide viewed from below along the axis. The outside pointing Sug*p* residues are indicated by red asterisks in panels (**D**,**E**).

The full-length trimeric structures of Orf39 and Gp298 were modeled using the multimer prediction functionality of AlphaFold3. The models provide a comprehensive overview of the tail fiber structures and indicate where the RBDs lie within the trimeric assemblies (Figure 9A,B). The structures of the predicted receptor-binding sites (Figure 7) in the Orf39 and Gp298 monomers were structurally aligned and are noticed to be structurally similar, consistent with the functional data showing shared receptor usage. The structural alignment of their binding sites is presented in Figure 9C.

The quality of the predicted structures was assessed using the pLDDT and the Predicted Aligned Error (PAE) tools. Both Orf39 and Gp298 models exhibited high average pLDDT scores (>80), which indicates a high degree of confidence in the local protein folds (Figure S9). Both trimeric structures have some disordered regions (pLDDT score < 60) in the termini and loop regions, along with the confident core structures. Furthermore, the PAE plots (Figure S10) showed low error values at the inter-chain interfaces, providing strong evidence for the predicted trimeric quaternary structure and validating the subunit interactions. Overall, Orf39 is predicted to be a highly stable, well-defined trimer with strong inter-chain confidence. Its low-confidence regions are restricted to minor, expected flexible loops. Gp298 is predicted to have a stable monomer structure, but a less certain trimer interface (yellow/orange PAE). Crucially, it contains distinctively disordered segments (red pLDDT), particularly at the N-termini (1–130 aa).

#### 3.5.3. The RBDs of Gp298 and Orf39

The experimental data (Section 3.2.3) and sequence analysis (Figure 7) predicted that Orf39 (residues 159–243) and Gp298 (residues 547–613) share a functional role in carbohydrate receptor binding. The corresponding residues in the previously described structural models indicated that the local structure of this region in Orf39 is extremely stable (Figure S9A). This domain presents a fixed, highly reliable binding surface with no predicted internal movement. Also, this segment locks the trimer together and is not flexible relative to the other chains (Figure S10A). The Orf39 trimer likely functions as a single, rigid scaffold. The binding sites on the 159–243 segments are presented in a fixed, highly precise spatial arrangement (e.g., three binding pockets fixed at specific distances) (Figure 9). This might be ideal for binding a specific, large, or ordered carbohydrate array where structural precision is paramount. The average pLDDT score of this region in Orf39 is 96.21, indicating it to be an extremely stable fold, being part of a primarily well-defined domain.

In case of the Gp298 (547–613) trimer fold, the predicted core fold is stable, but the yellow dips in the confidence plots suggest that small flexible loops are part of the binding surface or its immediate surroundings (Figure S9B). This segment forms a more dynamic trimer interface. The monomers can shift slightly relative to each other (Figure S10B). The average pLDDT value of this zone is 92.85, which also indicates this RBD in Gp298 to be a highly stable core with some flexible elements present.

Although Orf39 and Gp298 share significant sequence and structural similarity within their RBDs, the full trimeric assemblies were predicted to be quite different. A simple alignment of the full-length trimers is therefore misleading, as the overall size and architecture of the tail fibers differ beyond the receptor-binding site. Furthermore, the C-terminal tail region was predicted to be highly variable across different modeling runs due to its flexible nature, which complicates the interpretation of its specific conformation. This structural heterogeneity is consistent with the lower pLDDT scores observed in this region of the models.

#### 3.5.4. A Specific Carbohydrate Binding Cleft on the Orf39 Trimer Is Proposed by Molecular Docking

To generate a model and a hypothesis for the binding mechanism of the OC pentasaccharide ligand to Orf39, molecular docking was performed using AutoDock Vina. The docking utilized the monomeric RBD part (residues 159–243, as shown in Figure 9C,D) of the high-confidence, AlphaFold2-derived model of trimeric Orf39 (see Section 2.17.2). A blind docking approach was employed to search the entire RBD surface of the rigid Orf39 monomer for favorable binding pockets. The docking simulation generated 18 distinct binding poses (conformations), ranked by their predicted binding affinity (ΔG). The highest-scoring pose, Mode 1, yielded a binding affinity of ΔG = −6.378kcal/mol. However, this pose was dismissed because the pentasaccharide was oriented with the proximal Sug*p*-residue buried inside the Orf39 trimer, precluding it from being linked to the inner core heptose residue of the LPS [12]. On the contrary, this was not the case with the slightly lower-scoring Mode 2 ΔG = −6.273 kcal/mol) that was selected as the representative binding conformation. In this pose, the Sug*p* residue is positioned on the outside of the Orf39 trimer (Figure 9C,D). This pose was also noticed to consistently localize the binding site to a prominent inter-subunit cleft formed by the interface of two Orf39 monomer chains (Figure 9D). A summary of the top 18 binding affinities and the full search space defined for the blind docking is provided in the Supplementary Information (Table S1).

#### 3.5.5. Molecular Mechanism of Recognition: Backbone Anchoring and Hydrophobic Stabilization

**Figure 10:**
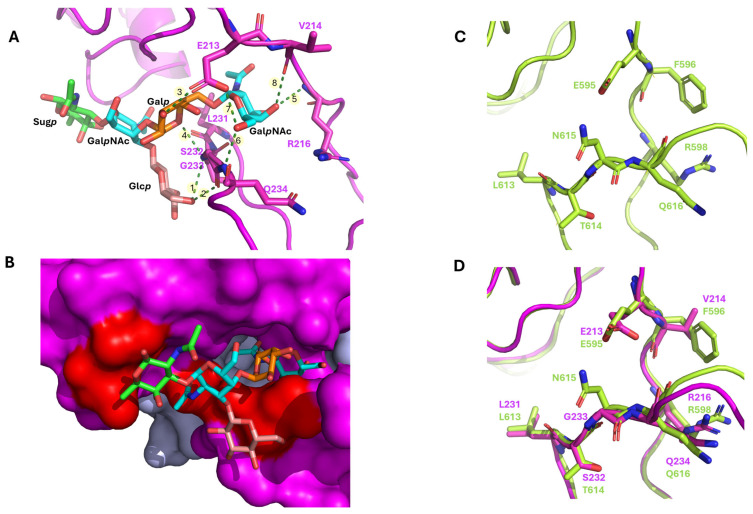
Atomic details of the protein-pentasaccharide interactions. (**A**). The OrfF39—pentasaccharide interaction zone is zoomed with the interacting residues of Orf39 shown in sticks. The pentasaccharide residues are colored as indicated in Figure 9. The Orf39 aa residue numbering is used for the aa residues. The hydrogen bonds indicated by green dashed lines are numbered according to Table S5. (**B**). The interaction zone of Orf39 is shown as a surface presentation. Visible are two Orf39 monomers in different colors, with violet showing the interacting Orf39 monomer and gray showing the adjacent monomer in the trimer. Note that the distal Gal*p*NAc residue of the pentasaccharide enters a cavity in the protein structure that should be able to accommodate the additional Glc*p* residue present in the OC structure but absent from the modeled pentasaccharide. The hydrophobic Orf39 surface aa residues interacting with the pentasaccharide (Table S5) are colored in red. (**C**). The predicted pentasaccharide interaction zone of Gp298 is shown in sticks. The residues are numbered using Gp298 residue numbering. (**D**). The pentasaccharide interaction zone of Orf39 (purple) aligned with that of the Gp298 (green).

The detailed structural analysis of the selected Mode 2 conformation, focusing on non-covalent interactions, identified a total of 13 contacts between the pentasaccharide and the Orf39 RBP (Table S5). The binding profile was a balance between stability derived from some Hydrophobic Contacts (HC) and structural specificity derived from eight Hydrogen Bonds (HB). The hydrophobic contacts were predominantly centralized around the terminal Gal*p*NAc residue, which was anchored deep within the pocket by residues including Glu213 and Arg216. This burying of the terminal sugar appears to contribute to the bulk of the binding stability (ΔG) (Figure 10B).

In contrast, the hydrogen bonds were critical for precise molecular recognition, primarily involving the Gal*p* residue (Figure 10A). Specifically, the backbone N-H of Gly233 formed a critical hydrogen bond with the O6 hydroxyl group of the Glc*p* residue, acting as the key structural anchor. The geometric details of these atomic interactions are summarized in Supplementary Table S5.

As anticipated, the respective binding pocket residues modeled for Gp298 RBD align perfectly with those of Orf39 RBD (Figure 10C,D) and reveal two significant substitutions that may explain the minor differences in the minimal receptor structures for φR1-37 and enterocoliticin. Gly233 in Orf39 is substituted by Asn615 in Gp298, and Val214, by Phe596 (Figure 10D).

## 4. Discussion

### 4.1. The Enterocoliticin Biosynthesis Gene Cluster

The 34,019 bp *Y. enterocolitica* strain 29930 genomic insert in Cos141 (Figure 2) is colinear with the chromosomal DNA of *Y. enterocolitica* 29930. A total of 42 predicted genes were identified that showed similarities to cryptic prophages in *Y. frederiksenii* ATCC 36641, *Y. kristensenii* ATCC 33638, *Y. mollaretii* ATCC 43969, *Y. bercovieri* ATCC 43970, *Y. enterocolitica* subsp. *enterocolitica* 8081 or *subsp. palearctica* 105.5R(r) and Y11, *Y. pseudotuberculosis* YPIII, IP 31758, pB1/+ and IP 32953, *Y. pestis* biovar Microtus str. 91001 and *Serratia symbiotica* str. Tucson. The predicted genes *orf6*–*orf24* and *orf30*–*orf40* may play a role in enterocoliticin biosynthesis. The presence of homologous sequences in other *Yersinia* species suggests the presence of a common precursor, supported by the production of phage tail-like bacteriocins by *Y. frederiksenii*, *Y. kristensenii* and *Y. intermedia* [61]. In general, numerous phage tail-like bacteriocins have been described in different gram-negative and gram-positive bacteria [24,62,63,64,65]. In addition, database searches using BlastX identified consistently cryptic phages in different *Yersinia* strains, and BlastN with the complete Cos141 sequence showed the presence of large regions of homologous DNA in *Y. pseudotuberculosis* strain YPIII (CP000950) and *Y. enterocolitica* O:9 strain 105.5R (CP002246) [66]. In the literature on these strains, there are no indications of the formation of phage-tail-like particles [66]; it can be assumed that these strains do not form them.

The application of highly purified enterocoliticin in a mouse model showed that in the gastrointestinal tract, there is only a short-term reduction in the *Yersinia* titer, and the bacterial numbers recovered rapidly after the treatment [67]. The large size of enterocoliticin may hinder its penetration into deeper tissues. A possibility to use, for example, the *E. coli* strain Nissle 1917 as a surrogate host for enterocoliticin expression could also be considered to reduce pathogen invasion [68]. However, the synthesis of enterocoliticin particles in *E. coli* VCS257/Cos141 was relatively low compared to the production of the particles by *Y. enterocolitica* 29930. An application using *E. coli* strains to deliver the particles in an animal host would require a different genetic construct for the expression of enterocoliticin particles.

The biotechnical production of enterocoliticin and the prophylactic application on animals in food production could help reduce the risk of infection due to the consumption of raw pork products. Similar experiments have been carried out with *Paenibacillus polymyxa* NRRL-B-30509 that produces a bacteriocin as an additive in chicken feed, resulting in a drastic reduction in *Campylobacter jejuni* bacteria counts in the chicken intestine [69]. Another possibility would be to add enterocoliticin to finished foods, comparable to the use of bacteriophage preparations to combat *Listeria monocytogenes* in food [70,71].

As enterocoliticin targets *Y. enterocolitica* serotype O:3, O:5,27 and O:9 strains [20] that comprise the dominant human pathogenic strains [72,73], so a therapeutic use of enterocoliticin to combat other *Y. enterocolitica* strains is conceivable as well [20,74].

### 4.2. φR1-37 RBP

Our work demonstrated that the tail fiber protein Gp298 of φR1-37 requires the chaperone protein Gp297 for trimerization, and very likely the Gp297 remains associated with the tail fiber after trimerization, as the two proteins could not be separated by chromatography (Figure 5C). This view is corroborated by the fact that Gp297 was also identified as a phage particle-associated protein (PPAP) [13]. Thus, we can only conclude that the Gp298-Gp297 complex forms the RBP of the phage. Interestingly, when searching homologous proteins to Gp297 in Cos141, the only hit was Orf40, i.e., encoded by the gene downstream of the Orf39 encoding gene, thus having a similar genomic order as that of *g298* and *g297* (Figure 4).

The Gp298-Gp297 complex was able to block the surface of the *Y. enterocolitica* YeO3-R1 (Figure 6C). Collectively, these results suggested that the tail fiber structure containing Gp298 and Gp297 is the part that interacts with the host receptor and plays a key role in binding. *Y. enterocolitica* YeO3-R1 showed a significant decrease in phage infectivity after Gp298–297 blocking. This means that most of the host receptor structures were inaccessible for phage φR1-37 as they were blocked by the receptor-bound tail fiber proteins.

It is worth noting that some phages use specialized phage-encoded chaperone proteins that aid in the assembly and disaggregation of supramolecular structures to properly fold and assemble tail fiber proteins [75,76,77,78,79,80]. Notable instances include the assembly of T-even phage tail fiber proteins such as Enterobacteria phages T4 and T2, which show the variability in chaperone-assisted assembly routes. For example, Gp38 has a different role in phage T2 and related phages (such as Enterobacteria phages T6, RB32, RB16, RB43, and RB49), compared to phage T4. In phage T2, an unrelated Gp38 product binds to a folded and trimeric Gp37, serving as the true adhesin [81,82,83]. The C-terminus of the folded Gp37 fiber is proteolytically cleaved and capped by Gp38, which likewise appears to be trimeric [84]. Our findings indicate that a similar scenario exists for the φR1-37 tail fiber.

### 4.3. In Silico Prediction of the Tail Fiber Protein Structure and Molecular Docking

#### 4.3.1. Structural Identity, Functional Homology, and Modular Evolution

The predicted trimeric architectures of both Orf39 and Gp298, generated using advanced deep-learning methods, provide a structural framework for understanding their function. The high pLDDT and iPTM scores lend confidence to these models and their predicted quaternary structures. Notably, the models suggest that despite global structural differences between the full tail fiber proteins, both Orf39 and Gp298 may possess a structurally closely similar carbohydrate-binding pocket within their homologous 80–85 amino acid RBDs. This structural similarity offers a plausible explanation for their functional homology—specifically, their shared ability to target the same bacterial surface receptor. Furthermore, the presence of this specific motif in diverse phage tail proteins is consistent with a modular evolutionary mechanism, where optimized binding domains may be horizontally transferred or adapted across different bacteriocins and phages. Variations in binding specificity are likely dictated by specific amino acid substitutions associated with the binding cleft. The alignment of the 80/85 aa domain hits (Figure S7) reveals that several signature residues in Orf39 and Gp298 are not conserved in other sequences, suggesting that these related proteins may have distinct binding specificities.

#### 4.3.2. Predicted Binding Energetics and Molecular Determinants of Recognition

The estimated low binding free energy (ΔG ≅ −6.3 kcal/mol) for the pentasaccharide docking to Orf39 trimer hints at the potential for a high-affinity receptor interaction. The Vina simulation predicted two distinct, low-energy binding pockets on Orf39, designated as Mode 1 and Mode 2 (Table S1). While both pockets represent theoretically possible interaction sites, Mode 2 was prioritized for further analysis with this specific oligosaccharide, as it appears to align more closely with the anticipated biological and spatial constraints of the tail fiber assembly.

Structural analysis of the predicted Mode 2 pocket suggests a possible binding mechanism where stability and specificity may be distributed across different components of the ligand. The terminal Gal*p*NAc residue is predicted to contribute to bulk stability through hydrophobic contacts, potentially helping to anchor the ligand within the inter-subunit pocket. Conversely, the specificity of the interaction could be mediated by the central Glc*p* and Gal*p* residues. These sugars are predicted to form highly constrained hydrogen bonds with specific residues in the RBD, most notably Gly233. Such interactions might represent the key determinant points for molecular recognition, potentially allowing the protein to distinguish its target carbohydrate array from other cell surface structures. The presence of two distinct predicted pockets in the simulation further suggests that the receptor-ligand interaction may be dynamic, potentially involving allosteric mechanisms or multiple binding states during the initial phases of host engagement. While our models provide a robust theoretical framework, empirical confirmation of the proposed RBP-receptor interactions will ultimately substantiate the anticipated value of this research.

#### 4.3.3. In Vivo Feasibility of the Binding

The prioritization of Mode 2 as a potentially relevant binding orientation is based on spatial and geometric considerations regarding the accessibility of the sugar residues in the OC hexasaccharide. In a physiological context, the Sug*p* residue—which links the hexasaccharide to the inner core—would likely remain accessible. Simultaneously, the distal residues (Gal*p*, Gal*p*NAc, and Glc*p*) must be positioned to interact with the tail fiber binding pocket. Our modeling suggests that Mode 2 may be the most plausible orientation to satisfy these steric requirements during initial contact.

**Figure 11:**
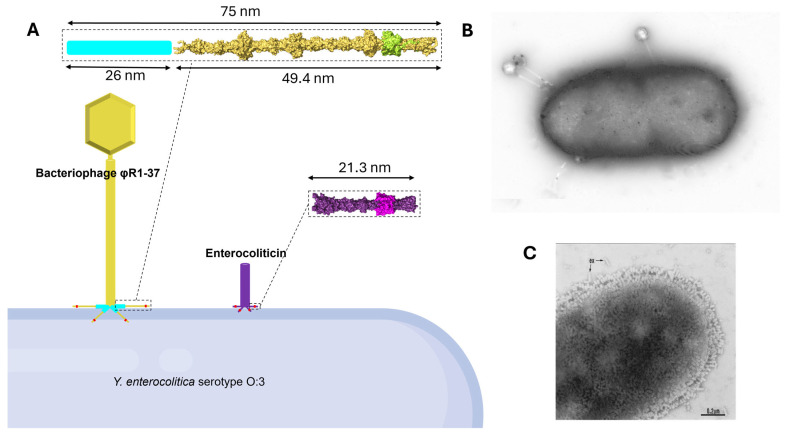
Phage and enterocoliticin tail fiber interactions with the bacterial surface. (**A**) Schematic illustration of the interactions of the tail fibers on the bacterial cell surface. The phage φR1-37 and enterocoliticin are drawn with the correct relative sizes using the same colors as in Figure 9. The phage φR1-37 tail fiber total length of 75 nm [13] means that it likely is connected to the tail with a proximal subunit of 26 nm (turquoise) in addition to the 49.4 nm Gp298. The phage height (head + neck + tail) is 450 nm, and the tail diameter is 15 nm. The enterocoliticin length is 80 nm, and the diameter is 15 nm [20]. The RBDs of the tail fibers are indicated with the red dots in the illustration. (**B**) Electron micrograph of phage φR1-37 adsorbed on the bacterial surface. The image was originally published in [10]. (**C**) Electron micrograph of enterocoliticin adsorbed on the bacterial surface. The image was originally published in [20]. The OC oligosaccharide is evenly distributed on the bacterial surface and covers ca. 70% of the bacterial surface, as indicated by the dense adsorption of enterocoliticin on it.

Furthermore, the rigid architecture of the tail fiber may impose physical constraints on host engagement. A vertical interaction appears unlikely, as a protein tip extending beyond the RBD would theoretically penetrate or interfere with the cell membrane (Figure 9B). Consequently, we propose a “low-profile” horizontal interaction, where the fiber lies parallel to the bacterial surface (Figure 11). While the trimeric assembly inherently provides three potential binding pockets, it is hypothesized that a single pocket may carry out the initial high-affinity interaction required for stable surface adhesion, representing a potentially thermodynamically more favorable scenario than simultaneous multi-pocket engagement.

## Figures and Tables

**Table 1:** Bacterial strains, plasmids and phages used in this study.

Bacteria	Strain	Serotype	Description (Reference)
*Y. enterocolitica*	YeO3-R1	O:3	Spontaneous rough derivative of YeO3-c, virulence plasmid cured (pYV-negative), φR1-37 sensitive [8]
	3229	O:50	Human stool isolate [25]
	18425/83	O:25,26,44	Human stool isolate [9]
	14779/83	O:5	Human stool isolate [25]
	29930	O:7,8	Food isolate [20]
	8081	O:8	Fatal septicemia [26]
	DSM 13030	O:3	DSMZ *
	13169	O:3	Pig, Free University of Berlin
	29807	O:5	Pig, Free University of Berlin
*Y. similis*	R708Ly	O:9	Isolated from a mole in Japan [9]
*Y. intermedia*	821/84	O:52,54	Human stool isolate [25]
*E. coli*	*BL21* Star		Expression system
	VCS 257		Stratagene
	DH5α		Stratagene
	GeneHogs		Stratagene
Plasmids	pCDF duet-1^TM^		Expression vector, lac promoter, 2 MCS, Strept^R^ (Novagen)
	pCP-1		The φR1-37 gene *g298* cloned to pCDF duet-1^TM^ at MCS-1
	pCP-2		The φR1-37 gene *g297* cloned to pCP-1 at MCS-2
	SuperCos1		Amp^R^, Neo^R^
Phage	φR1-37		[9]

* Leibniz Institute DSMZ-German Collection of Microorganisms and Cell Cultures.

**Table 2:** Inhibitory activity of supernatants of *E. coli* VCS257/Cos141 against different *Y. enterocolitica* strains. Activity units were obtained by serial dilutions of culture supernatants after filter sterilization (SF) and after concentration in a CsCl-gradient ultracentrifugation (UCG). n.d., no detectable inhibition.

Tested Strain	Serotype	SF *	UCG *
*Y. enterocolitica* 13169	O:3	100	6400
*Y. enterocolitica* DSM13030	O:3	400	6400
*Y. enterocolitica* 8081	O:8	n.d.	n.d.
*Y. enterocolitica* 29807	O:5	n.d.	n.d.

* inhibitory growth activity (AU per mL).

**Table 3:** EOPs of φR1-37 on different *Yersinia* strains.

Bacterial Species	Strain	Serotype	EOP
*Y. enterocolitica*	YeO3-R1	O:3	1
*Y. enterocolitica*	3229	O:50	0.83 × 10^−2^
*Y. similis*	R708Ly	O:9	0.5
*Y. intermedia*	821/84	O:52,54	0.5
*Y. enterocolitica*	18425/83	O:25,26,44	0.16 × 10^−7^
*Y. enterocolitica*	14779/83	O:5	0.16 × 10^−8^

## Data Availability

The Cos141 genomic insert was submitted to Genbank under accession number PV390083. The WGS sequence data (raw sequences and assemblies) were deposited at NCBI with accession codes BioProject ID PRJNA1241681 and BioSample ID SAMN47560904. The protein/carbohydrate structural data that support the findings of this study has been deposited in the data base Zenodo (https://zenodo.org/records/18589322, accessed 10 February 2026).

## Supplementary Materials

The following supporting information can be downloaded at: https://www.mdpi.com/article/10.3390/v18030291/s1. Supplementary Figures: Figure S1: Analysis of the enterocoliticin-associated structural proteins; Figure S2: DOC-PAGE gel with LPS isolated from the *Yersinia* strains mentioned in Table 2; Figure S3: BlastP search hits for Gp298; Figure S4: BlastP search hits for enterocoliticin protein Orf39; Figure S5: BlastP search hits for Orf39 85 aa domain; Figure S6: BlastP search hits for GP298 80 aa domain.; Figure S7: Alignment of receptor binding site aa sequences of selected BLASTp hits; Figure S8: Structural models of the tail fiber proteins and the predicted receptor binding domain.; Figure S9: Per-residue confidence plots for the AlphaFold3-predicted structures of the Orf39 and Gp298 trimers; Figure S10: Predicted Aligned Error (PAE) matrix for the AlphaFold3-predicted trimeric structure of Orf39 and Gp298; Supplementary Tables: Table S1: Complete List of Predicted Binding Poses from AutoDock Vina. Table S2: PCR primers used for the screening of the cosmid library; Table S3: Annotation of Cos141 sequence; Table S4: Identification of MALDI-TOF-MS peptides in proteins of the Cos141 sequence. Table S5: The molecular details of the pentasaccharide-Orf39 atomic-level interactions.

## Abbreviations

The following abbreviations are used in this manuscript:

aa	amino acid
Amp	Ampicillin
API	application programming interface
AU	Activity unit
BLAST	Basic local alignment tool
BV-BRC	Bacterial and Viral Bioinformatics Resource Center
Clm	Chloramphenicol
DB	Dynabeads
DOC	Deoxycholate
ECL	Enhanced chemiluminescence
EOP	Efficiency of plating
Gal*p*	d-galactose
Gal*p*NAc	*N*-acetyl-d-galactosamine
Glc*p*	d-glucose
HB	Hydrogen Bond
HC	Hydrophobic Contact
HPLC	High pressure liquid chromatography
ipTM	interface predicted TM-score
IPTG	Isopropyl-β-d-1-thiogalactopyranoside
Kan	Kanamycin
KmR	Kanamycin-resistance
LA	Lysogeny broth agar
LB	Lysogeny broth
LPS	Lipopolysaccharide
LTF	Long tail fiber
MALDI	Matrix-assisted laser desorption/ionization
MCS	Multicloning site
MS	Mass spectrometry
MW	Molecular weight
OC	Outer core
OD	Optical density
OPS	O-polysaccharide
Orf	Open reading frame
PAE	Predicted aligned error
PAGE	Polyacrylamide gel electropohoresis
PBS	Phosphate-buffered saline
PCR	Polymerase chain reaction
PFU	Plaque forming unit
PGAP	Prokaryotic Genome Annotation Pipeline
PFF	peptide fragment fingerprint
pLDDT	Predicted local distance difference test
PLIP	Protein-Ligand Interaction Profiler
PMF	Peptide mass fingerprint
PPAP	phage particle associated protein
PVDF	polyvinylidene difluoride
RASTtk	Rapid annotations using subsystems technology tool kit
RBD	Receptor Binding Domain
RBP	Receptor Binding Protein
RT	Room temperature
SDS	Sodium dodecyl sulphate
SF	Filter sterilization
Str	Streptomycin
Sug*p*	2-acetamido-2,6-dideoxy-d-*xylo*-hex-4-ulopyranose
TBST	Tris-buffer with sodium chloride and tween 20
TEM	Transmission electron microscopy
TEMED	Tetramethylethylenediamine
TOF	Time of flight
UCG	CsCl gradient ultracentrifugation
WGS	Whole genome sequencing
