# Supplementary material for: Identification of Receptor Binding Proteins of Yersinia Phage φR1-37 and Enterocoliticin That Use the Same Bacterial Surface Receptor
Source: Viruses. 2026 Feb 27;18(3):291. doi: 10.3390/v18030291 (PMC13030884; doi:10.3390/v18030291)
Supplement: Supplementary file 1 [file viruses-18-00291-s001.zip › viruses-4106077-supplementary.pdf]

viruses

Supplementary Information

Identification of receptor binding proteins of *Yersinia* phage φR1-37 and enterocoliticin that use the same bacterial surface receptor

Mikael Skurnik [1,*], Rahime Tetik [2], Muhammad Suleman Qasim [1,3], Jana Sachsenröder [2], Ralf Dieckmann [2,4], Carlos G. Leon-Velarde [5], Göran Widmalm [6], Eckhard Strauch [2] and Arnab Bhattacharjee [1,7]

1 Human Microbiome Research Program, Department of Bacteriology and Immunology, Faculty of Medicine, 00014 University of Helsinki, Helsinki, Finland; muhammad.qasim@helsinki.fi (M.S.Q.); arnab.bhattacharjee@helsinki.fi (A.B.)
2 Department Biological Safety, German Federal Institute for Risk Assessment, 10589 Berlin, Germany; ra_rahime@yahoo.de (R.T.); jana.sachsenroeder@bfr.bund.de (J.S.); dieckmannr@rki.de (R.D.); eckhard.strauch@bfr.bund.de (E.S.)
3 RNAcious Laboratory, Molecular and Integrative Biosciences Research Programme, Faculty of Biological and Environmental Sciences, 00014 University of Helsinki, Helsinki, Finland
4 Department 1—Infectious Diseases, Robert Koch Institute, 13353 Berlin, Germany
5 Laboratory Services Division, University of Guelph, Guelph, ON, N1H 8J7, Canada; cleonvel@uoguelph.ca
6 Arrhenius Laboratory, Department of Chemistry, Stockholm University, S-106 91 Stockholm, Sweden; goran.widmalm@su.se
7 Drug Research Program, Division of Pharmaceutical Biosciences, Faculty of Pharmacy, 00014 University of Helsinki, Helsinki, Finland
* Correspondence: mikael.skurnik@helsinki.fi

1. Supplementary Figures

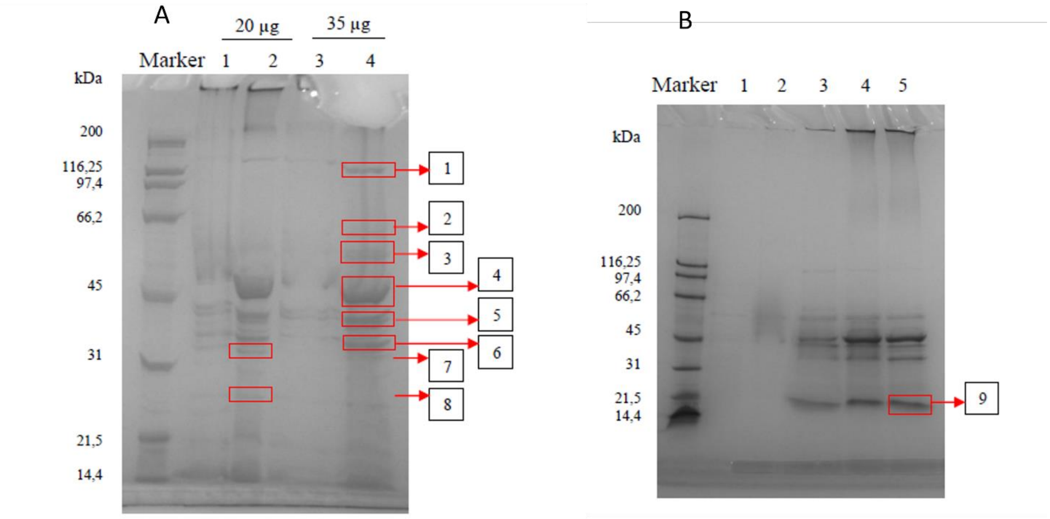

Figure S1: Analysis of the enterocoliticin-associated structural proteins. **A)** SDS-PAGE for the separation of enterocoliticin proteins in 12.5% precast gel after the CsCl density gradient (1 and 3) and subsequent dialysis (2 and 4); application of 20 µg (1 and 2) or 35 µg (3 and 4). Broad range marker (Bio-Rad Laboratories, Munich, Germany). **B**) SDS-PAGE for the separation of enterocoliticin proteins in 20% precast gel. Application of 20 µg enterocoliticin sample after sterile filtration (1), after ultra centrifugation (2), after 1st CsCl gradient (3) with subsequent dialysis (4) and second CsCl-gradient purification followed by ultrafiltration (5). The red boxes indicate the protein bands that were cut out for MALDI TOF MS analysis.

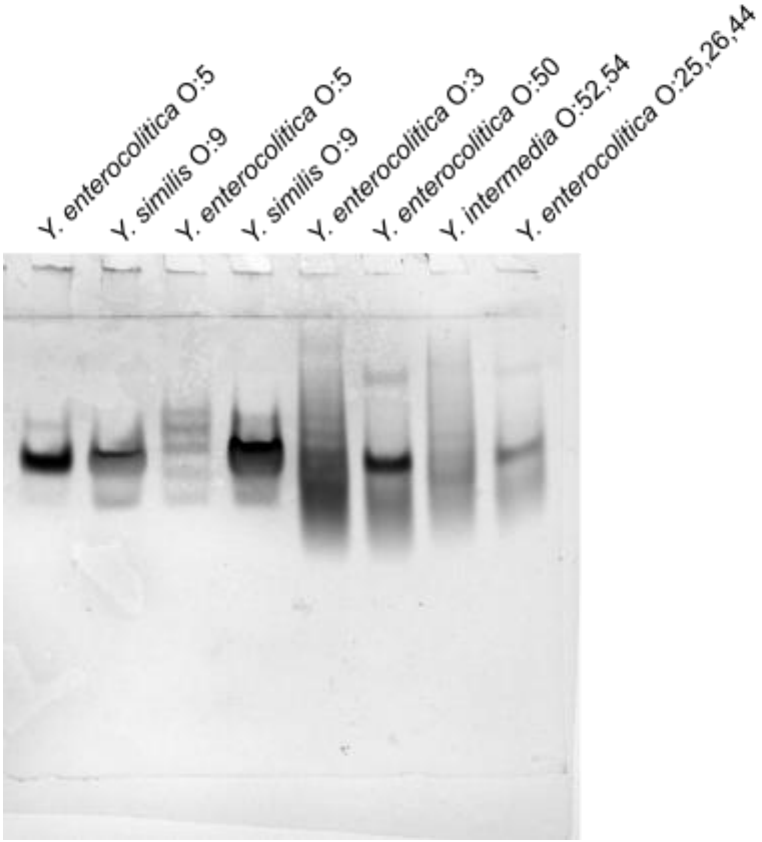

Figure S2. DOC-PAGE gel with LPS isolated from the *Yersinia* strains mentioned in **Table 2**.

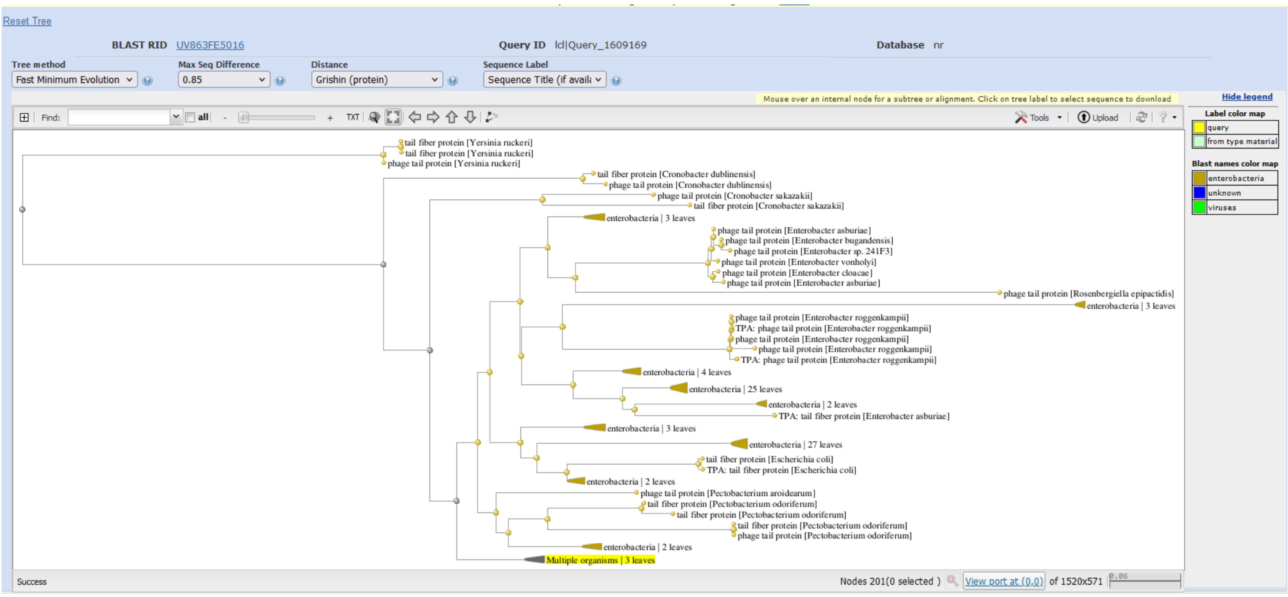

Figure S3. BlastP search hits for Gp298.

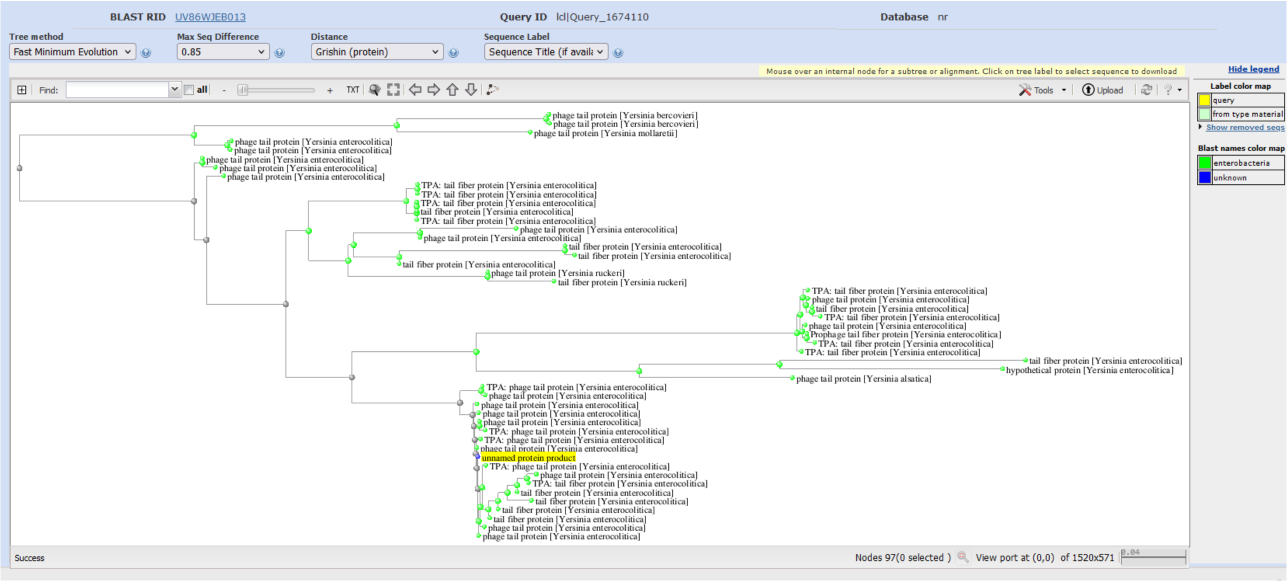

Figure S4. BlastP search hits for enterocoliticin protein Orf39.

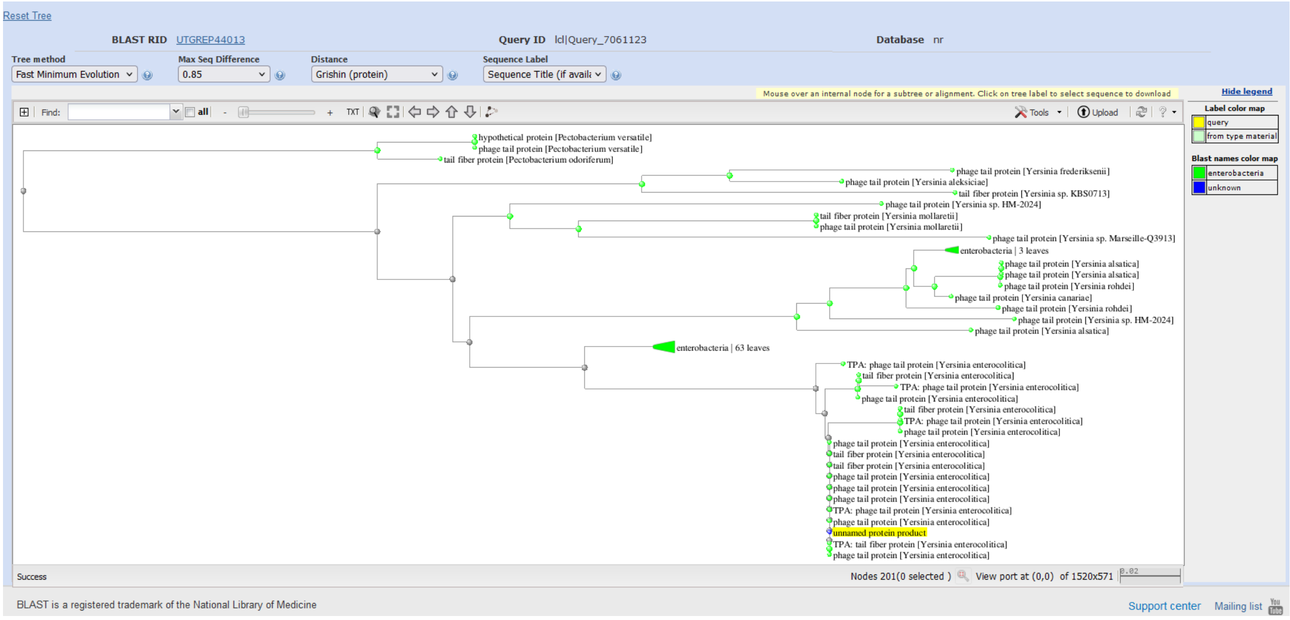

Figure S5. BlastP search hits for Orf39 85 aa domain.

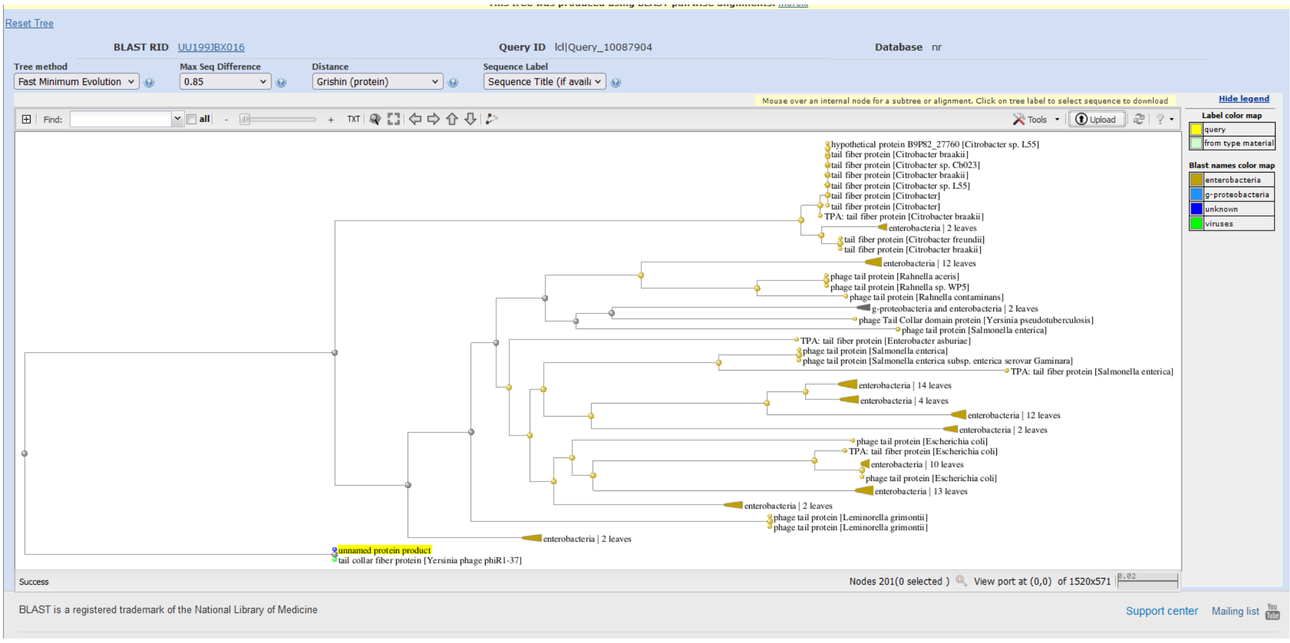

Figure S6. BlastP search hits for Gp298 80 aa domain.

Gp298 80 aa hits

```
YP_004934532      PIGVPIPYPLATPPKNFLKANGSAFDQVAFPKLLNYYPSGVLPDLRGEFIRGWDDGRNVDTGRNILTNQGDAIRNIT  Gp298
WP_153681183      PVGVPVPYPLATPPTGWMKCNGSSFNLASYPKLAAVFPSGVLPDLRGEFIRGWDDGRNVDSGRQLLSQQGDTIQNIT
WP_181039645      PVGVPIPYPLATPPAGFLKLNGASFDKAKYPRLAAIYPGGVLPDLRGEFIRGWDDGRGADAGRNLLSYQGDAIRNIT
HAY5209043        PVGVPVPYPLATPPTGWMKCNGSSFNLASYPKLAAVFPSGVLPDLRGEFIRGWDDGRNVDSGRQLLSQQGDTIQNIT
WP_250951981      PVGVPVPYPLATPPTGWMKCNGSSFNLASYPKLAAVFPSGVLPDLRGEFIRGWDDGRNVDSGRQLLSQQGDTIQNIT
MCV5274177        PVGVPVPWPSATPPTGWLKCNGAAFSAVEYPKLARVYPTNKLPDLRGEFIRGWDDGRGIDAGREILSAQGDAIRNIT
EOJ7624023        PVGVPVPWPLETPPTGWLKCNGAAFSSEKYPKLAKAYPTNKLPDLRGEFIRGWDDGRGVDAGRQLLSSQGDAIRNIE
STM65058          PVGVPVPWPSATPPTGWLKCNGAAFSAEEYPELAKAYPTNKLPDLRGEFIRGWDDGRGVDSGRTILNSQGDAIRNIT
WP_085049731      PVGFPLPWPQATPPGGWLKCNGATFDKAKYPKLATAYPSGVLPDLRGEFLRGWDDGRGVDVGRQILTAQGDAIRNIT
WP_235814458      PVGAPVPYPSATPPAGWIKCNGQSFNKSAYPQLATAYPSGVLPDLRGEFVRGWDDGRGADAGRTLLSAQGDAIRNIT
EMD7330430        PVGVPVPWPSATPPTGWLKCNGAPFSAEEYPKLAKVYPTNELPDLRGEFIRGWDDGRGIDAGREILSAQGDAIRNIT
HEA3226513        PVGIPVPWPSATPPTGWLKCNGSPFNLASYPKLAAVFPSGVLPDLRGEFIRGWDDGRGVDAGRAILTSQTDALQQIT
MFK8361351        PVGVPVPWPSATPPTGWLKCNGAAFSAEEYPELAKAYPTNKLPDLRGEFIRGWDDGRGMDTGRAILSAQGDAIRNIY
WP_181621256      PVGFPLPWPQATPPGGWLKCNGATFDKVKYPKLATAYPSGVLPDLRGEFLRGWDDGRGVDSGRALLSSQSDAIRNIT
```

Orf39 85 aa hits

```
EKN6076146      PVGIPLPYPLADIPAP-5-WFKMNGGSFNTTTYPKLAAKYPTGVLPDLRGEVIRGWDDGRGVDVSRVLLSGQLDAIRNITGGF  Orf39
WP_230079026    PVGIPLPYPLADIPAP-5-WFKMNGGSFNTTTYPKLAAKYPTGVLPDLRGEVIRGWDDGRGVDVSRVLLSGQLDAIRNITGGF
WP_268215876    PVGIPLPYPLADILAP-5-WFKMNGGSFNTTTYPKLAAKYPTGVLPDLRGEVIRGWDDGRGVDVSRALLSGQLDAIRNITGGF
WP_432340291    PIGIPIPYPLANIPAT-5-FFKMNGGSFNVTTYPKLAAKYPTGVLPDLRGEVIRGWDDERGIDVNRALLSGQLDAIRNITGGF
WP_054878754    PIGIPLPYPLADIPAT-5-FFKMNGGSFNMTTYPKLAVKYPSGVLPDMRGNVIRGWDDGRGVDTGRALLSEQLDALQKITGSI
HGC0667836      PVGVPVPYPLATPPTG   WMKCNGSSFNKTLLPALAAVYPSGVLPDLRGEFIRGWDDGRGVDVGRALMSAQGDAMRNISGTV
WP_087795985    PVGIPLPWSTATPPAG   WIKCNGNSFNKTTYPKLALAYPSGVLPDLRGEFIRGWDDGRGADAARTLLSAQSDAMRNLTGSF
WP_089078668    PVGVPVPYPLATPPTG   WMKCNGSSFNKTLLPALAAVYPSGVLPDLRGEFIRGWDDGRGVDVGRALMSAQGDAMRNISGTV
MBN3096478      PVGVPIPYPSATPPTG   WIKCNGQSFNKTTYPQLAGAYPSGILPDLRGEFVRGWDDGRGADAGRGLMSAQGDAIRNITGSL
HDL8284792      DIGIPIPYPLASVPAG   YLKCNGATFSTSTYPKLALKYPSGVLPDLRANAIRGWDDGRGVDAGRVLLSQQDDAIRNITGIY
WP_258876668    PVGAPVPYPSATPPAG   WIKCNGQSFNKSAYPQLATAYPSGVLPDLRGEFVRGWDDGRGVDTGRGLLSAQSDAIRNITGEF
WP_254911723    DIGIPIPHPLASVPAG   YLKCNGATFSTSTYPKLALKYPSGVLPDLRANAIRGWDDGRGVDAGRVLLSQQDDAIRNITGTF
EPB4725214      PVGVPVPWPSATPPAG   WLKCNGAPFTTSQYPKLATVYPSGVLPDLRGEFLRGWDDGRGIDVERAILSSQGDAIRNITGKF
WP_230857911    PIGIPLPYPLATPPAG   FLKVNGSSFSTTTYPKLALAYPSGVLPDMRGNAIRAWDDGRGIDAGRALLSEQLDALQNITGNF
HAY5209043      PVGVPVPYPLATPPTG   WMKCNGSSFNLASYPKLAAVFPSGVLPDLRGEFIRGWDDGRNVDSGRQLLSQQGDTIQNITGEV
```

Figure S7. Alignment of receptor binding site aa sequences of selected BLASTp hits. These hits were selected from the highest, middle and lowest scores among the 100 hits. The top sequences in both alignments are the query sequences. The aa residues conserved in all sequences are highlighted in grey. The signature residues in Orf39 and Gp298 identified from the Orf39 docking experiments are indicated in red.

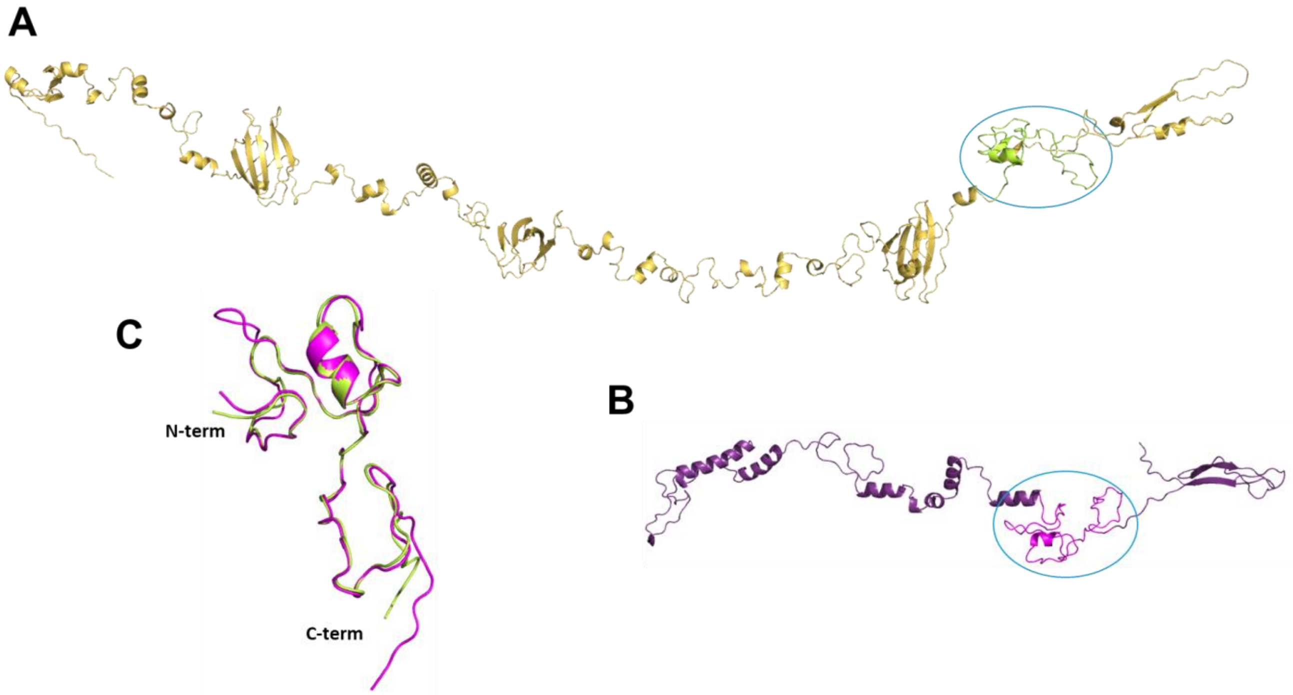

Figure S8. Structural models of the tail fiber proteins and the predicted receptor binding domain. **A**. Gp298. **B**. Orf39. **C**. Structural alignment of the receptor binding domains of Gp298 (lime) and Orf39 (magenta).

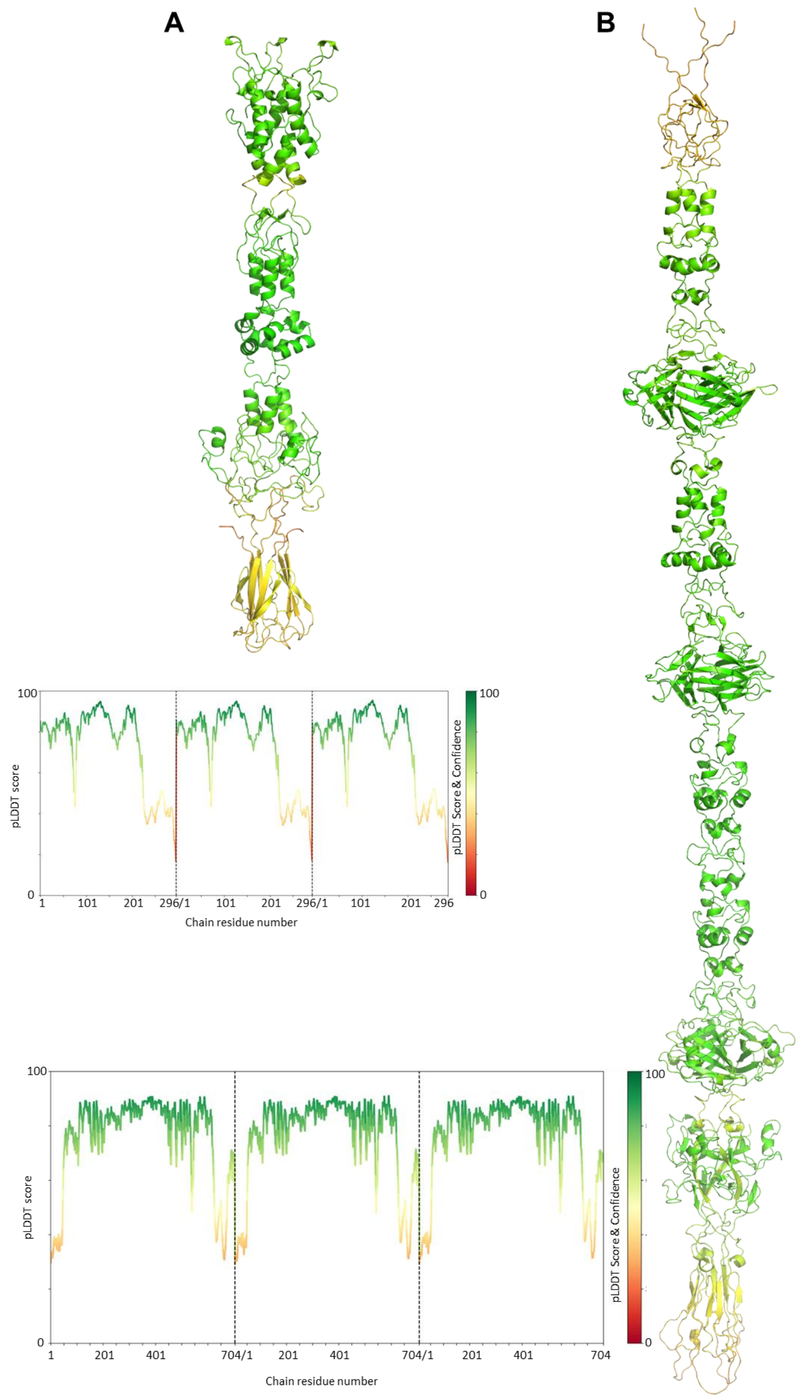

Figure S9. Per-residue confidence plots for the AlphaFold3-predicted structures of the Orf39 (**A**) and Gp298 (**B**) trimers. The plot displays the predicted local distance difference test (pLDDT) score for every residue in the full protein assembly. The x-axis represents the sequential residue numbers for the three chains in the modelled assembly. The y-axis represents the pLDDT score, ranging from 0 to 100. The pLDDT metric estimates the confidence in the local structure (the Cα trace) of each residue, independent of its orientation relative to other chains. The plot is coloured according to the standard AlphaFold confidence scheme: The very high confidence (green): pLDDT≥90 is typically found in structured domains; the predicted structure is highly reliable, potentially comparable to experimental structures. The high confidence (yellow): 70≤pLDDT<90 is generally a reliable backbone prediction for most secondary structures. The low confidence (orange): 50≤pLDDT<70 often indicates unstructured regions (e.g., loops or flexible linkers) where the backbone trace is predicted, but the exact side-chain positions may be inaccurate. The very low confidence (red): pLDDT<50 typically represents regions predicted to be highly disordered or unfolded, where the model has essentially no confidence in the local structure.

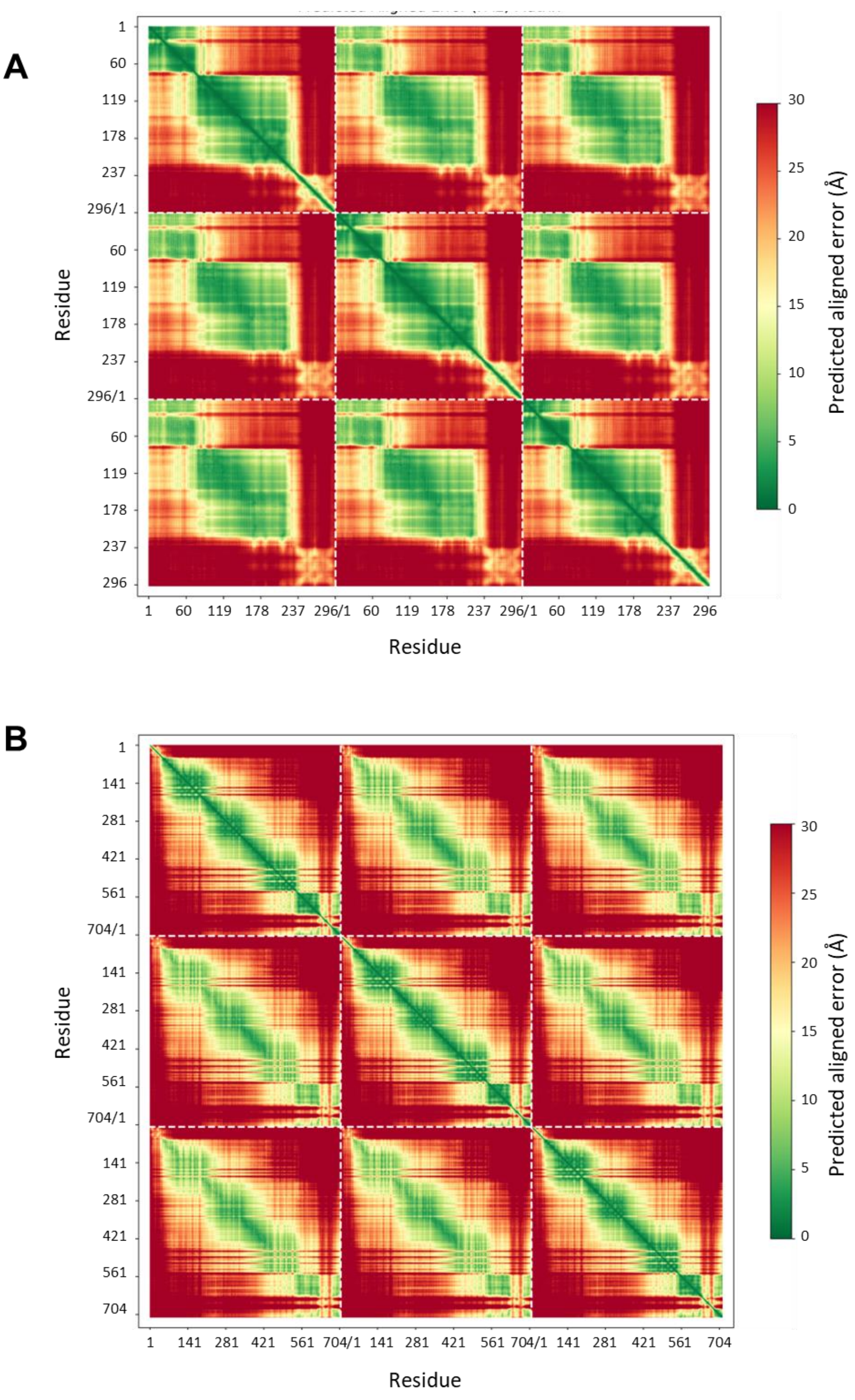

Figure S10. Predicted Aligned Error (PAE) matrix for the AlphaFold3-predicted trimeric structure of Orf39 (**A**) and Gp298 (**B**). The PAE plot is a heatmap where the colour at position (i,j) represents the expected Cα distance error (in Å) between the predicted position of residue i and the predicted position of residue j, after optimal alignment of the structure. The x- and y-axes span the sequential residue numbers of the full trimeric assembly. The assembly is structured as chain A, followed sequentially by chain B, and then chain C. Colour values follow a gradient: green (0 Å) that signifies extremely high confidence (low expected error) in the relative alignment, while red (≥15 Å) indicates low confidence (high expected error). The key interpretations are as follows: The diagonal blocks show the intra-chain confidence (the predicted distance error between residues within the same monomer). Off-diagonal blocks show the inter-chain confidence (the predicted distance error

between residues belonging to different chains, reflecting the reliability of the trimeric interface and quaternary structure). Green colouring in the off-diagonal regions indicates a high-confidence, stable trimeric assembly.

2. Supplementary Tables

Table S1. Complete List of Predicted Binding Poses from AutoDock Vina with the 18 lowest-energy binding poses (modes) identified during the molecular docking of the pentasaccharide to the Orf39 RBP trimer. The binding affinity (ΔG) and structural divergence (RMSD) from the globally best mode (Mode 1) are reported for each pose. The high RMSD values for Modes 2, 3, 5, 7, and 9 indicate the presence of multiple, geometrically distinct binding locations. **Mode 2** was chosen as the final representative complex.

Mode	Affinity (**ΔG** kcal/mol)	RMSD (Å) from Best Mode (Mode 1)	RMSD (Å) Upper Bound
1	−6.378	0.00	0.00
2	−6.273	13.36	16.97
3	−6.237	12.74	19.66
4	−6.138	3.507	7.915
5	−6.046	13.43	16.89
6	−5.922	1.83	4.90
7	−5.875	16.27	19.88
8	−5.753	4.381	9.011
9	−5.698	15.65	19.22
10	−5.615	15.60	19.16
11	−5.592	16.48	20.08
12	−5.534	16.21	19.83
13	−5.488	13.92	17.51
14	−5.412	12.90	16.48
15	−5.350	14.10	17.66
16	−5.291	1.871	4.908
17	−5.215	14.70	18.30
18	−5.180	15.82	19.41

Table S2. PCR primers used for the screening of the cosmid library

**Primer**	**Sequence 5'-3'**	**Position in Cos141**	**Size of PCR product**
Tailshif3	CCAGACTGAGCAGCAAAACTGTG	9978-10000	
Tailshif4	TTGGCACCGTCGCATTCAAGAC	9084-9105	916 bp

Table S3. Annotation of Cos141 sequence. Two annotations are shown. Annotation of gene products according to GenBank (BlastP search against Protein db) and RAST tool kit (RASTtk) of the Genome Annotation Service of the Bacterial and Viral Bioinformatics Resource Center (BV-BRC)

Gene	Start	End	Size (bp)	Strand	Size (aa)	BLASTP annotation	RASTtk annotation
orf1	1	585	585	-	195	MFS transporter	Putative transport protein
orf2	975	1988	1014	+	337	MBL fold metallo-hydrolase	Hypothetical protein
orf3	2058	3845	1788	-	595	Methyl-accepting chemotaxis protein	Methyl-accepting chemotaxis sensor/transducer protein
orf4	4172	5107	936	-	311	Omptin family outer membrane protease	Protease VII (Omptin) precursor (EC 3.4.23.49) @ Outer membrane protease Pla
orf5	5272	5514	243	-	80	DinI family protein	Stress response protein
orf6	5763	6485	723	-	240	LexA family transcriptional regulator	Phage repressor protein cI
orf7	6760	7239	480	+	159	Antiterminator Q family protein	Phage antitermination protein Q
orf8	7504	7782	279	+	92	Hypothetical protein	FIG027828: Putative bacteriophage protein
orf9	7819	8148	330	+	109	DUF2570 domain-containing protein	Probable phage antitermination protein Q
orf10	8201	8368	168	+	55	Hypothetical protein	Hypothetical protein
orf11	8454	8984	531	+	176	ATP-binding protein	Phage tail sheath protein
orf12	8989	9183	195	+	64	DUF2635 domain-containing protein	Phage protein Mup38
orf13	9180	10688	1509	+	502	Phage tail sheath subtilisin-like domain-containing protein	Phage tail sheath Mup39, L
orf14	10756	11124	369	+	122	Phage tail tube protein	Phage tail tube protein Mup40, M
orf15	11126	11425	300	+	99	Phage tail assembly protein	Phage protein
orf16	11546	12901	1356	+	451	Chemotaxis protein	Phage tape measure protein Mup42
orf17	12989	14389	1401	+	466	DNA circularization N-terminal domain-containing protein	Phage tail/DNA circulation protein Mup43, N
orf18	14386	15456	1071	+	356	Contractile injection system protein, VgrG/Pvc8 family"	Phage tail protein Mup44, P
orf19	15472	16068	597	+	198	Phage baseplate assembly protein	Phage baseplate assembly protein Mup45, Q
orf20	16065	16517	453	+	150	Phage GP46 family protein	Phage protein Mup46, V
orf21	16521	17657	1137	+	378	Baseplate J/gp47 family protein	Phage protein Mup47, W
orf22	17654	18250	597	+	198	YmfQ family protein	Phage protein Mup48
orf23	18298	19353	1056	+	351	Tail fiber protein	Phage tail fiber Mup49, S
orf24	19364	19834	471	+	156	Tail fiber assembly protein	Hypothetical protein
orf25	19993	20733	924	+	307	MurR/RpiR family transcriptional regulator	Transcriptional regulator, RpiR family
orf26	20900	22441	1542	+	513	PTS transporter subunit EIIC	PTS system, β-glucoside-specific IIB or IIC component
orf27	22530	23966	1437	+	478	6-phospho-beta-glucosidase	6-phospho-β-glucosidase (EC 3.2.1.86)
orf28	24242	24976	735	+	244	Carbonic anhydrase	Carbonic anhydrase, alpha class (EC 4.2.1.1)

orf29	25286	26317	1032	-	343	Methyltransferase	O-methyltransferase, family 2
orf30	26781	27074	294	+	97	Phage tail assembly protein	Phage protein
orf31	27319	28404	1086	+	361	Transglycosylase SLT domain-containing protein	Phage tape measure protein Mup42
orf32	28430	28696	267	+	88	Hypothetical protein, lytic domain	Phage tail length tape-measure protein
orf33	28736	29134	399	+	132	Hypothetical protein	Hypothetical protein
orf34	29204	29371	168	+	55	Hypothetical protein (pseudogene)	Phage tail/DNA circulation protein
orf35	29375	29533	159	+	52	Baseplate J/gp47 family protein (pseudogene)	Phage protein Mup47, W
orf36	29633	29836	204	-	67	Baseplate J/gp47 family protein (pseudogene)	Phage protein Mup47, W
orf37	30001	30255	255	+	84	Baseplate J/gp47 family protein	Phage protein Mup47, W
orf38	30451	30837	387	+	128	YmfQ family protein	Phage protein Mup48
orf39	30887	31777	891	+	296	Phage tail protein	Phage tail fiber Mup49, S
orf40	31836	32255	420	+	139	Tail fiber assembly protein	FIG01222416: Hypothetical protein
orf41	32472	33455	984	-	327	Acyltransferase family protein	Hypothetical protein
orf42	33808	34017	210	+	70	Recombinase family protein (pseudogene)	Phage DNA invertase

Table S4. Identification of MALDI-TOF-MS peptides in proteins of the Cos141 sequence (RASTtk annotation)

Band	Estimated size (kDa)	Identified peptides	Cos141 match		Predicted function	Calculated MW (Da) *
			Gp	Location		
1	116	(R)ADINGEFTDGNVAGGVPPTILPAEWFNTLQR(E)	Orf23	18328:18426	Phage tail fiber Mup49	36578
2	66	(R)TPLFFAEFDNSQANTASTTQR(T)	Orf13	9216:9284	Phage tail sheath Mup39	53089
		(K)ITVTTPAAATGVISLYIGGIR(V)		9489:9557		
		(R)FAAGSAIITPNVIR(A)		10446:10493		
		(R)IFAVLNQFR(L)		10641:10673		
		(M)TIPFTNIPSNLR(T)		9180:9221		
		(R)WSYSQQLYGHSFAAQSGTYGQLTAAGELR(N)		9948:10040		
		(R)LAEFGFSSGLPAGSDVVIGFLGGDR(S)	Orf19	15619:15699	Phage baseplate assembly protein Mup45	21267
3	55	(R)IFAVLNQFR(L)	Orf13	10641:10673	Phage tail sheath Mup39	53089
		(M)TIPFTNIPSNLR(T)		9180:9221		
		(R)FAAGSAIITPNVIR(A)		10446:10493		
		(R)VQIELTLIDGR(T)	Orf16	12794:12832	Phage tape measure protein Mup42	49276
4	45	(M)TIPFTNIPSNLR(T)	Orf13	9180:9221	Phage tail sheath Mup39	53089
		(R)FAAGSAIITPNVIR(A)		10446:10493		
		(K)ITVTTPAAATGVISLYIGGIR(V)		9489:9557		
		(R)TPLFFAEFDNSQANTASTTQR(T)		9216:9284		
		(R)WSYSQQLYGHSFAAQSGTYGQLTAAGELR(N)		9948:10040		
		(R)IFAVLNQFR(L)		10641:10673		
		(R)VDVLWTGVLINQLR(I)		10599:10646		
5	40	(K)FTLNWGETAQAVIEHVTR(W)	Orf18	14815:14874	Phage tail protein Mup44	39259
		(R)WAALLYYDQPDGHLYLTR(V)		14869:14928		
6	35	(K)IGDIPNTR(A)	Orf23	18304:18333	Phage tail fiber Mup49	36578
		(R)ADINGEFTDGNVAGGVPPTILPAEWFNTLQR(E)		18328:18426		
7	34	(K)LFGIGGQSAAYFIR(V)	Orf22	17939:17986	Phage protein Mup48	21877
8	25	(K)ALGYSITVTQYR(Q)		17990:18031		
		(K)LFGIGGQSAAYFIR(V)		17939:17986		
		(R)AVINSYELTR(Q)	Orf11	8733:8768	Phage tail sheath protein	19732
9	20	(K)ETVVAPSISCTIR(D)	Orf14	10900:10944	Phage tail tube protein Mup40	12832
		(R)LAGTAYVTVDGITVMVAGQFK(Y)		10774:10842		

* MWs were calculated using Expasy (https://web.expasy.org/peptide_mass/)

viruses

MDPI

Table S5. The molecular details of the pentasaccharide-Orf39 atomic-level interactions. This is shown for Mode 2 summarizing the complete non-covalent interaction profile with a total of 13 unique contacts between the pentasaccharide ligand and the Orf39 RBP monomer. The hydrogen bond and hydrophobic contact interactions are indicated. The protein residue numbers refer to the full-length Orf39 sequence. The pentasaccharide residues and their proximal (reducing end residue 1) to distal order (terminal residue 5) is indicated (see main text figure 8). The hydrogen bond numbers refer to the main text Figure 10

Sugar residue	Order	Orf39 residue	Protein Atom	Ligand Atom	Distance (Å)	Bond number
Hydrogen bonds						
Glc*p*	3	Gly 233	Backbone N	O6	3.33	1
Glc*p*	3	Gly 233	Backbone C=O	O6	2.70	2
Gal*p*	4	Glu 213	Sidechain OE1	O3	2.83	3
Gal*p*	4	Gly 233	Backbone N	O5	3.19	4
Gal*p*NAc	5	Arg 216	Backbone N	O4	3.14	5
Gal*p*NAc	5	Gln 234	Backbone N	O6	2.92	6
Gal*p*NAc	5	Glu 213	Sidechain OE2	O6	2.95	7
Gal*p*NAc	5	Val 214	Backbone C=O	O4	2.70	8
Hydrophobic interactions						
Sug*p*	1	Thr 204	Cβ	C6	3.71	
Sug*p*	1	Val 206	Cγ	C5	3.59	
Glc*p*	3	Ser 232	Cα	C2	3.71	
Gal*p*NAc	5	Gly 233	Cα	C5	3.59	
Gal*p*NAc	5	Leu 231	Cδ	NAc	3.54	
